# A Novel Gene *SbSI-2* Encoding Nuclear Protein from a Halophyte Confers Abiotic Stress Tolerance in *E. coli* and Tobacco

**DOI:** 10.1371/journal.pone.0101926

**Published:** 2014-07-07

**Authors:** Narendra Singh Yadav, Vijay Kumar Singh, Dinkar Singh, Bhavanath Jha

**Affiliations:** 1 Discipline of Marine Biotechnology and Ecology, CSIR-Central Salt and Marine Chemicals Research Institute, Bhavnagar, Gujarat, India; 2 Academy of Scientific and Innovative Research, CSIR, New Delhi, India; University of Delhi South Campus, India

## Abstract

*Salicornia brachiata* is an extreme halophyte that grows luxuriantly in coastal marshes. Previously, we have reported isolation and characterization of ESTs from *Salicornia* with large number of novel/unknown salt-responsive gene sequences. In this study, we have selected a novel salt-inducible gene *SbSI-2* (*Salicornia brachiata salt-inducible-2*) for functional characterization. Bioinformatics analysis revealed that SbSI-2 protein has predicted nuclear localization signals and a strong protein-protein interaction domain. Transient expression of the RFP:SbSI2 fusion protein confirmed that SbSI-2 is a nuclear-localized protein. Genomic organization study showed that *SbSI-2* is intronless and has a single copy in *Salicornia* genome. Quantitative RT-PCR analysis revealed higher *SbSI-2* expression under salt stress and desiccation conditions. The *SbSI-2* gene was transformed in *E. coli* and tobacco for functional characterization. pET28a-SbSI-2 recombinant *E. coli* cells showed higher tolerance to desiccation and salinity compared to vector alone. Transgenic tobacco plants overexpressing *SbSI-2* have improved salt- and osmotic tolerance, accompanied by better growth parameters, higher relative water content, elevated accumulation of compatible osmolytes, lower Na^+^ and ROS accumulation and lesser electrolyte leakage than the wild-type. Overexpression of the *SbSI-2* also enhanced transcript levels of ROS-scavenging genes and some stress-related transcription factors under salt and osmotic stresses. Taken together, these results demonstrate that *SbSI-2* might play an important positive modulation role in abiotic stress tolerance. This identifies *SbSI-2* as a novel determinant of salt/osmotic tolerance and suggests that it could be a potential bioresource for engineering abiotic stress tolerance in crop plants.

## Introduction

The world population is increasing rapidly and may reach 6 to 9.3 billion by the year 2050, whereas crop production is decreasing rapidly because of the negative impact of various environmental stresses; therefore, it is now very important to develop stress tolerant varieties to cope with this upcoming problem of food security [1]. Major abiotic stresses includes high salinity, drought, temperature extremes, water logging, high light intensity, and mineral deficiencies. Abiotic stresses reduce plant growth and development, causing poor productivity or plant death in extreme conditions. Abiotic stresses are the primary causes of crop loss worldwide, reducing average yields of major crop plants by more than 50% [2]. Plants adapt to environmental stresses via a plethora of responses, including the activation of molecular networks that regulate stress perception, signal transduction and the expression of both stress-related genes and metabolites. Plants have stress-specific adaptive responses as well as responses which protect the plants from more than one environmental stress [3]. Various genes induced by abiotic stresses are grouped under two categories, namely functional genes and regulatory genes. The first category of genes generally facilitates production of important enzymes and metabolic proteins, which include osmolytes, transporters/channel proteins, antioxidative enzymes, lipid biosynthesis genes, polyamines and sugars. The second category of genes consists of regulatory proteins, such as Transcription factors (TFs) belonging to the bZIP, DREB, MYC/MYB, and NAC families, which control expression of many downstream stress tolerance genes [4], [5], [6]. A number of abiotic stress-related genes, as well as some transcription factors and regulatory sequences in plant promoters have been characterized [4], [7]. Whole genome sequencing and microarray analysis have provided valuable insight towards the understanding of molecular mechanism of abiotic stress tolerance involving a number of functional and regulatory genes [7], [8]. Transcription factors modulate expression of specific groups of genes through sequence specific DNA binding and protein-protein interaction. They can act as activators or repressors of gene expression, leading to specific cellular responses. Abiotic stress related TFs follow ABA dependent and independent pathways. Identification of key regulatory TFs and their regulatory activators and repressors, their target genes and protein partners is essential to understand the regulatory complex networks. Studies in *Arabidopsis* and *Oryza sativa* indicated that a number of *cis*-elements and their corresponding binding proteins, i.e. TFs, are involved in plant stress responses [4]. It has been reported that transgenic plants overexpressing genes encoding key transcription factors showed enhanced tolerance to various abiotic stresses [6], [9], [10], [11], [12], [13].

Halophytes are useful organisms to study salt tolerance mechanisms because they are well adapted to salinity and can overcome this problem more efficiently than glycophytes [14]. Halophytes have a unique genetic makeup allowing them to grow and survive under salt stress conditions [15]. Experimental studies in our laboratory have concentrated on an extreme halophyte, *Salicornia brachiata* Roxb., in an effort to identify and characterize novel/unknown genes that enable salt tolerance. *S. brachiata* (*Amaranthaceae*), a leafless succulent annual halophyte, commonly grows in the salt marshes of Gujarat coast in India. *Salicornia* can grow in a wide range of salt concentrations (0.1–2.0 M) and can accumulate quantities of salt as high as 40% of its dry weight [15]. This unique characteristic provides an advantage for the study of salt tolerance mechanisms. Therefore, this plant may serve as a potential bioresource for salt-responsive genes study.

Expressed sequence tags (ESTs) analysis is a rapid and powerful method for elucidating information regarding gene expression and also provides an opportunity to identify new genes involved in biological functions. EST databases have been developed in many glycophytic plant species in response to different stresses like cold, desiccation, high salinity and ABA [2], [16] and also in some halophytes. In addition to known functional genes, unknown and hypothetical genes provide a good candidate pool to find novel stress tolerance genes. A large number of unknown genes, which lack similarity with known genes in the NCBI database, have been reported. It can be envisaged that these unknown genes constitute the unique genetic make-up of the plant helping it to sustain itself under stress condition. EST databases of different halophytic plants show a large percentage of unknown genes like *Sueda salsa* (22%, [17]), *Mesembryanthum crystallinum* [18], *Thellungiella halophilla* (32%, [19]), *Avicennia marina* (30%, [20]), *Limonium sinense* (37%, [21]), *Aleuropus littoralis* (20%, [22]), *Spartina alterniflora* (13%, [23]), *Macrotyloma uniflorum* (30%, [24]), *S. brachiata* (29%, [25]), *Tamarix hispida* (21%, [26]), Alfalfa (22%, [27]) and *Chenopodium album* (42%, [28]). Previously, we have identified approximately 1000 ESTs in response to salt stress from *S. brachiata* [25] and also characterized some important abiotic stress tolerant genes (*SbGST*, [29]; *SbMAPKK*, [15]; *SbDREB2A*, [30]; *SbNHX1*, [31]; *SbASR1*, [32]; *SbSOS1*, [33]; *SbSI-1*, [34]). The *S. brachiata* EST database contains large number of novel/unknown/hypothetical genes [25]. These genes might be playing an important role in providing salinity stress adaptation to *Salicornia*, and therefore can serve as a valuable bioresource for engineering abiotic stress tolerance in crop plants. With this aim we have characterized a novel salt-inducible gene *SbSI-2* in response to salt and osmotic stress, through its heterologous expression in *E. coli* and tobacco.

## Materials and Methods

### Ethics statement

Plant samples were collected from open coastal areas. Locations are not the part of any national parks or protected areas, thus do not require any specific permits. It is further to confirm that the field studies did not involve endangered or protected species.

### Plant growth and stress treatments

*Salicornia brachiata* seeds were harvested from dried plants collected from the coastal area near Bhavnagar (Latitude 21° 45′N, Longitude 72° 14′E), Gujarat, India. The seeds were germinated in plastic pots containing garden soil and the plants were grown in natural conditions. One-month-old seedlings were carefully uprooted and transferred to hydroponic culture (½ major and minor MS stock, [35]) in a culture room with a dark/light cycle of 8/16 h at 25°C for one month. The nutrient solution was renewed twice in a week. Plants were given different stress treatments like salt stress (250 mM NaCl) and desiccation by wrapping in tissue paper for 0, 6, 12 and 24 h. Upon completion of the treatments, shoot tissues were collected, frozen in liquid nitrogen and stored at −80°C.

### Cloning of *SbSI*-2 gene

The EST of *SbSI-2* was made full length and characterized for its role in abiotic stress tolerance. Total RNA was extracted from salt stressed plants of *S. brachiata* by GITC method [36]. The 5′-RACE reaction was performed according to manufacturer's protocol (Invitrogen, San Diego, CA, USA). The first strand of cDNA was synthesized with a gene-specific primer GSP R1 (5′-TGATAATACATCCGGGCAGTT-3′) and Superscript RT II. The mRNA was removed with RNase H, and a homopolymeric tail was added to 3′-end of the cDNA. The dC-tailed cDNA was subjected to PCR amplification with gene specific primer GSP R2 (5′-ACCCCTGCATCTATCAACTCTG-3′) and an AAP (Abridged Anchor primer) primer (5′-GGCCACGCGTCGACTAGTAC(G)_16_-3′) supplied with kit. Further, nested PCR amplification was performed using a nested, gene-specific primer GSP R3 (5′-AGGGTTAGGGCAAGAAAGAAAG-3′) and AUAP primer (5′-GGCCACGCGTCGA CTAGTAC-3′) supplied with kit. The amplicon was purified from agarose gel and cloned into the pGEM-T Easy vector system II (Promega, Madison, Wisconsin) and sequenced (Macrogen Inc., Seoul, South Korea).

To perform 3′-RACE reactions, the first strand of cDNA was synthesized using PK1(oligo dT primer) adapter primer (5′- CCAGTGAGCAGAGTGACGAGGACTCGAGCTCA AGC(T)_17_ -3′). Following synthesis of the first strand of cDNA, PCR was performed with a gene-specific primer, GSP F1 (5′-AACTGCCCGGATGTATTATCAC-3′), and an adaptor primer, PK2 (5′-CCAGTGAGCAGAGTGACG-3′). Further, a nested PCR was setup by gene specific primer GSP F2 (5′-AAGGAAGCTCTTCTGGAGTTGA -3′) and an adaptor primer PK3 (5′-GAGGACTCGAGCTCAAGC-3′). The nested amplified fragments were purified from agarose gel and cloned into the pGEM-T Easy vector system II and sequenced.

The *SbSI-2* EST and the 5′- and 3′-RACE reactions were sequenced, and contiguous sequences were assembled to obtain the full-length *SbSI-2* gene. After determining the open reading frame, the full length *SbSI-2* cDNA was PCR-amplified with AccuPrime Pfx DNA polymerase (Invitrogen) in conjunction with SbSI2F (5′- CGC GGATCCATGGGATTTCATTCCTTTG -3′) and SbSI2R (5′-CCGGAATTCTCAACAAAT CGAATGAAGAA-3′) primers, containing *BamH1* and *EcoRI* sites, respectively. The amplification product was then cloned into a pJET1.2/blunt cloning vector (MBI Fermentas) and sequenced (Macrogen Inc., Seoul, South Korea).

### *In silico* analysis

The NCBI database was used as a search engine for nucleotide and protein sequences. TMpred online software was used for the prediction of transmembrane domains and ClustalW for sequence alignment. Indication of conserved domains of SbSI-2 gene was obtained by BLASTp (http://www.ncbi.nlm.nih.gov). Secondary structure prediction was carried out by Expasy tools (http://www.expasy.ch/tools/). Nuclear localization signals (NLS) of SbSI-2 protein were predicted by the WoLF pSORT [37] and CELLO Prediction server (http://cello.life.nctu.edu.tw/cgi/main.cgi). Discriminate score for being a nuclear protein, calculated from the presence of NLS motif, pat4, pat7, bipartite motif, and the amino acid composition [38]. Leucine-rich nuclear export signals (NES) were predicted by NetNES 1.1 server (http://www.cbs.dtu.dk/services/NetNES/) using a combination of neural networks and hidden Markov models. Protein-protein interaction domains were detected by PROFisis PredictProtein server (https://www.predictprotein.org/). Phosphorylation motifs were predicted by NetPhosK 1.0.

### Isolation of *SbSI*-2 genomic clone

Genomic DNA from *Salicornia* plant was isolated using CTAB-DNA extraction method [39]. PCR was conducted to amplify the *SbSI*-2 genomic fragment using SbSI2F and SbSI2R primers, which were used to amplify the complete open reading frame of *SbSI*-2 from the cDNA clone. The amplicon was gel purified, cloned in pGEM-T Easy vector and sequenced.

### Copy number analysis of *SbSI-2* gene by southern blotting

Southern blotting was performed to determine *SbSI-2* gene copy number. Genomic DNA (20 µg) from *S. brachiata* was digested with *EcoRI*, *HindIII*, and *SmaI* separated by electrophoresis (0.8% agarose gel) and transferred onto a Hybond N+ membrane (Amersham Pharmacia, UK) using alkaline transfer buffer (0.4 N NaOH with 1 M NaCl). Blot was hybridized with PCR generated probe for *SbSI-2* gene labeled with DIG-11-dUTP, following the manufacturer's user guide (Roche, Germany). Pre-hybridization and hybridization were carried out at 42°C overnight in DIG EasyHyb buffer solution (Roche, Germany). The hybridized membrane was detected by using CDP-Star chemiluminescent as substrate, following manufacturer user guide (Roche, Germany) and signals were visualized on X-ray film after 30 min.

### Subcellular localization of SbSI-2 protein

A translational fusion of SbSI-2 with RFP (red fluorescent protein) was made using Gateway technology [40]. The full length *SbSI-2* cDNA was PCR-amplified with AccuPrime Pfx DNA polymerase in conjunction with SbSI2CAF (5′-CACCATGGGATTTCATTCCTTTG-3′) and SbSI2CAR (5′-TCAACAAATCGAATGAAGAA-3′) primers. The blunt-end PCR product was then cloned into a pENTER/D-TOPO Entry vector (Invitrogen, USA) and sequenced. Thereafter, the LR recombination reaction was performed between an attL-containing Entry clone pENTER/D-TOPO-SbSI2 vector and an attR-containing destination vector pSITE-4CA by Gateway LR Clonase II enzyme mix (Invitrogen, USA). The resulting LR reaction was used to transform *E. coli* DH5α cells. Colonies growing on streptomycin containing media were checked for insertion of *SbSI-2* gene in Destination vector by PCR amplification. The resulting fusion construct (expression clone) was isolated and insertion of gene was confirmed through sequencing. The fusion construct (RFP:SbSI-2) was transferred into onion epidermal cells by particle bombardment with gene gun (PDS-1000/He Biolistic, Biorad, USA). The pSITE-4CA (RFP) vector was used as control. After incubation on MS plate for 12–24 h, the onion epidermal cells were observed for transient expression of RFP with an epifluorescence microscope (Axio Imager, Carl Zeiss AG, Germany). DAPI staining used as standard control for nuclear localization.

### Quantitative RT-PCR analysis

Total RNA was isolated from control and treated plant samples from *S.brachiata* using GITC method [36] and quantified using ND-1000 spectrophotometer (Nanodrop technologies, USA). The cDNA was prepared using 5 µg total RNA by Superscript RT III first-strand cDNA synthesis kit (Invitrogen, San Diego, CA). Real-time qPCR was performed on a Bio-Rad IQ5 detection system (Bio-Rad, U.S.A.) with QuantiFast Kit (Qiagen, USA). The PCR reactions was carried out containing 5 pmol of gene specific primers (forward 5′-CCCAGAAAGAAAAAGGCAAGA-3′ and reverse 5′-CTCCAGAA GAGCTTCCTTTGC-3′) and β-tubulin (forward 5′-GGAGTCACCGAGGCAGAG-3′ and reverse 5′-ATCACATATCAGAAACCACAA-3′) at 95°C-5 min followed by 95°C-10 s, 60°C-30 s and 72°C-30 s for 40 cycles, and continued for melting curve analysis to check the specificity of PCR amplification. The amplified product was resolved on a 1% agarose gel to check specificity of PCR product. Experiments were repeated twice independently. Fold changes were calculated using the CT method. CT values for individual variants were compared to CT values for a reference control (β-tubulin) for all treated samples and data was analysed using untreated plants at every time point as baseline control [41].

The expression patterns of reactive oxygen species (ROS) related genes (*NtSOD*, *NtAPX*, *NtCAT*) and some stress-responsive TFs (*NtDREB2* and *AP2*-domains containing TF) were also analyzed by qRT-PCR in both transgenic and WT plants after salt stress. Gene-specific primer pairs of ROS-related genes (*NtSOD*, *NtAPX* and *NtCAT*; primers sequence taken from Huang et al. [42]) and stress-responsive TFs *NtDREB2* (DREB2F 5′-GCCGACGCTAAGGATA TTCA-3′ and DREB2R 5′-TGCAAAACAGAGCTTCCTCA-3′) and *AP2*-domains containing TF (AP2dF 5′-AAGGGCGAGGAAGAACAAAT-3′ and AP2dR 5′-GTGGCTCTGGAA AGTTGA-3′) were utilized for expression studies, whereas QACTF (5′-CGTT TGGATCTTGCTGGTCGT-3′) and QACTR (5′-CAGCAATG CCAGGGAACATAG-3′) primers were used for actin. qRT-PCR reactions were carried out as described above and repeated three times to ensure reproducibility.

### Cloning of *SbSI-2* cDNA in pET28a expression vector and recombinant protein expression

The *SbSI-2* gene was excised from pJET1.2-SbSI-2 vector using *BamH1* and *EcoR1* restriction endonucleases and cloned in pET28a vector. *E. coli* BL21 (DE3) cells were transformed with recombinant plasmid (pET28a-SbSI-2) or pET28a vector alone. The recombinant protein was expressed by adding 1 mM IPTG at 0.6 OD_600_. Recombinant protein production was induced after 2 h of treatment and reached maximum at 6 h.

### Functional validation of *SbSI-2* gene in *E. coli* BL21 (DE3) cells under salt and desiccation stresses

#### Spot assay

A spot assay was carried out to ascertain the function of *SbSI-2* in *E. coli* cells. BL21 (DE3) cells were transformed with recombinant plasmid (pET28a-SbSI-2) and vector alone. Cells were grown in LB medium to 0.6 OD_600_. Thereafter, 1 mM IPTG was added and cells were grown for 12 h at 30°C. Next day cultures were diluted to 0.6 OD_600_, and then diluted to 10^−3^, 10^−4^ and 10^−5^. Ten microliters from each dilution was spotted on LB basal plates or supplemented with 500 mM NaCl, 500 mM KCl or 600 mM Mannitol.

#### Liquid culture assay

Functional analysis was also carried out in liquid culture using LB basal medium, as well as supplemented with NaCl, KCl, PEG and mannitol. *E. coli* BL21 (DE3) cells with recombinant plasmid or vector alone were grown as mentioned above, diluted to 0.6 OD_600_ and 400 µl cells were inoculated in 10 ml LB medium containing 500 mM NaCl, 500 mM KCl, 10% PEG (6000) and 600 mM mannitol, and incubated at 30°C. The bacterial suspension was harvested at every 2 h till 12 h and OD_600_ was measured.

### Construction of plant transformation vector and tobacco transformation

To perform plant transformation, *SbSI-2* cDNA was PCR-amplified with AccuPrime Pfx DNA polymerase in conjunction with SbSI2PF (5′-TCCGAGCTCATGGGATTTCATTCCTTTG-3′) and SbSI2PR (5′-CGCGGATCC TCAACAAATCGAATGAAGAA-3′) primers, which contained *SacI* and *BamHI* sites, respectively. The *SbSI-2* gene was cloned into the pRT100 vector [43] to add the *35S* promoter and terminator. The pRT100 plant expression vector contains strong and constitutive *35S* promoter from cauliflower mosaic virus and ampicillin resistance gene for bacterial selection. The *SbSI-2* amplicon was digested with *SacI* and *BamHI* restriction endonucleases. The pRT100 vector was also linearized by using the same set of restriction endonucleases and ligated overnight with *SacI/BamHI* digested *SbSI-2* at 8°C, which places *SbSI-2* under the control of *CaMV 35S* promoter and polyadenylation signal. The recombinant pRT100 vector was further transformed in *E. coli* DH5α cells. Colonies growing on ampicillin containing media were checked for insertion of *SbSI-2* gene by PCR amplification using gene specific primers. The above gene construct (pRT100-SbSI-2) was digested with the double cutter restriction enzyme *PstI* to get the entire expression cassette containing the *CaMV 35S* promoter, the *SbSI-2* gene, and the terminator. Thereafter, the entire expression cassette (35S-SbSI-2-terminator) was cloned into the pCAMBIA2301 binary vector at the *PstI* site. The resulting vector was mobilized into *Agrobacterium tumefaciens* (EHA 105) and used to transform tobacco (*Nicotiana tabacum* cv. Petit Havana) plants according to a standard protocol [44]. Putative transgenic plants regenerated directly from leaf edges in the presence of kanamycin were transferred to culture bottles that contained MS basal medium supplemented with kanamycin (100 mg/l). Transgenic lines were screened via GUS assay and PCR amplification analysis. Seeds of the selfcrossed transgenic lines were harvested for subsequent experiments.

### Confirmation of putative transgenic tobacco plants

#### Confirmation by PCR analysis

Genomic DNA was isolated from different lines via the CTAB (N-cetyl-N,N,N-trimethylammonium bromide) method [39]. To verify the presence of the transgene, PCR was conducted with gene-specific primers and *gus*-specific primers (gusAF 5′-GATCGCGAAAACTGTGGAAT-3′ and gusAR 5′-TGAGCGTC GCAGAAC ATTAC-3′). PCR products were analyzed on 0.8% agarose gel with appropriate size DNA marker.

#### Confirmation by histochemical GUS staining

GUS activity was visualized in leaf tissue with a β-glucuronidase Reporter Gene staining kit (Sigma, USA). Seedlings from transgenic plants were dipped into GUS staining solution, vacuum infiltrated for 2 min and then incubated overnight at 37°C in the dark. The tissues were then rinsed with 80% ethanol for 4 h to overnight to remove chlorophyll.

#### Confirmation of transgene integration and determination of copy number

Transgene integration and copy number was determined by Southern hybridization; for this, 20 µg of genomic DNA from each transgenic lines and WT plants were digested with *Hind III*. Digested DNA fragments were separated on 0.8% agarose and blotted onto a Hybond (N^+^) membrane (Amersham Pharmacia, UK) using alkaline transfer buffer (0.4 N NaOH with 1 M NaCl). A DIG-11-dUTP labeled gene specific DNA probe was synthesized by PCR according to the manufacturer's protocol (Roche, Germany). Hybridization was carried out at 42°C overnight in DIG EasyHyb buffer solution (Roche, Germany). The hybridized membrane was detected using CDP-Star as substrate (Roche, Germany) and signals were visualized on X-ray film.

#### *SbSI-2* transgene expression analysis by semi-quantitative RT-PCR

To check the mRNA levels of overexpressed *SbSI-2* gene in transgenic plants, semi-quantitative RT-PCR was carried out. Total RNA was isolated from WT and transgenic plant samples using GITC buffer and was quantified with a ND1000 spectrophotometer (Nanodrop Technology, USA). The cDNA was prepared using 5 µg of total RNA with a SuperScript RT III first-strand cDNA synthesis kit. The synthesized cDNA (1 µl, diluted 1∶5) was used as a template, and actin was used as an internal control for RT-PCR analysis. The *SbSI-2*-specific primer pair, RTF (5′- CCCAGAAAGAAAAAGGCAAGA-3′) and RTR (5′-CTCCAGAAGAGCTTCCTTTGC-3′), was utilized for expression study of the *SbSI-2*, whereas QACTF (5′-CGTTTGGATCTTGCTGGTCGT-3′) and QACTR (5′-CAGCAATG CCAGGGAACATAG-3′) primers were used for actin. PCR reactions were carried out in 1× PCR buffer supplemented with 200 µM dNTPs, 1.25 U Taq DNA polymerase and 5 pmol of each of the gene-specific primers according to the following conditions: an initial denaturation at 95°C for 3 min, 25 cycles at 94°C for 30 s, 60°C for 30 s and 72°C for 30 s, followed by a final extension at 72°C for 7 min. RT-PCR experiments were repeated three times, and the amplified products were analyzed via agarose gel electrophoresis.

### Evaluation of transgenic plants exposed to salt and osmotic stress

T_1_ transgenic lines were confirmed by PCR amplification with gene specific primers and *gus*-specific primers. To analyze the stress tolerance of *SbSI-2*-overexpressing tobacco plants, seeds were germinated in MS medium supplemented with 0, 200 mM NaCl (salt stress) and 300 mM mannitol (osmotic stress) in culture room conditions. The percentage of seed germination was scored 18 days after seed inoculation. T_1_ seedlings were also analyzed for different growth parameters under salt and osmotic stresses. At eight days, kanamycin-positive seedlings were transferred to MS medium supplemented with 0, 200 mM NaCl or 300 mM mannitol in petri dishes. Shoot length, root length, leaf surface area, fresh weight (FW), dry weight (DW) and relative water content (RWC) of the seedlings were measured after 30 days for salt and osmotic stress. Seedling tissues were collected after 45 days of salt and osmotic stress and subjected to various physiological and biochemical analyses. To study the growth for longer duration, the WT and transgenic lines seeds were first germinated on the MS basal medium, and after one week of germination, the kanamycin-positive seedlings were transferred in jars on the MS basal medium or supplemented with 200 mM NaCl.

#### Chlorophyll estimation

Seedling chlorophyll content of transgenic and WT plants, grown under different stresses, were estimated according to Arnon [45] and chlorophyll content was calculated per gram of fresh tissue weight [46].

#### Electrolyte leakage

Electrolyte leakage was measured as described by Lutts et al. [47].

#### Measurement of proline content

Free proline content in the seedlings was determined using acid ninhydrin as previously described by Bates et al. [48]

#### In vivo localization of O_2−_ and H_2_O_2_ content in the transgenic seedlings

*In vivo* detection of O_2_^−^ and H_2_O_2_ was accomplished by histochemical staining with nitro blue tetrazolium (NBT) and 3,3′- diaminobenzidine (DAB) as described by Shi et al. [49].

#### Na^+^, K^+^ and Ca^2+^ ion content analysis

Ion content was determined via the method described by Shukla et al. [50].

### Statistical analyses

Each experiment was performed three times with three replicates and data from fifteen plants were recorded in each replicates. One-way ANOVA between subject factors was performed by ezANOVA (http://www.cabiatl.com/mricro/ezanova/) for analysis of variance to determine the least significant difference between means. Mean values that were significantly different at p≤0.05 within treatment from each other are indicated by different letters (a, b and c).

## Results

### Isolation and sequence analysis of *SbSI-2* cDNA

Previously, we have identified about 1000 ESTs in response to salt stress, from the extreme halophyte *Salicornia brachiata*. Among these, *SbSI-2* EST (Gen-Bank accession number EB485109) was made full length using the 5′ and 3′ RACE. The *SbSI-2* cDNA (Gen-Bank accession number JX872273) was 537 bp long, contained a 26 bp 5′ UTR, a 423 bp open reading frame and an 88 bp 3′ UTR region (Fig. S1A). The cDNA encoded a polypeptide of 140 amino acid residues with a predicted molecular mass of 15.93 kDa and an isoelectric point of 10.34. *SbSI-2* did not reveal homology with known gene by NCBI protein blast analysis and showed matching with unknown/hypothetical genes. Hydropathicity analysis by TMpred program (http://www.ch.embnet.org/cgi-bin/TMPRED_form_parser) showed that SbSI-2 has no transmembrane domains. The secondary structure of SbSI-2 was analyzed by PSIPRED protein structure prediction software showed that peptides contain 2 alpha helixes, 5 extended strands and 8 random coils (Fig. S1B).

ScanProsite (http://au.expasy.org/) has revealed three distinct regions (1–60, 61–120 and 121–140) based on 3 distinct amino acids sequence patterns (Fig. S2). The WoLF PSORT program revealed nuclear localization signals (Fig. S3 A) in SbSI-2 protein. A possible cleavage site is located between amino acids 24 and 25. The SbSI-2 protein contained three pat4 motifs, two pat7, two bipartite motifs showing NLS Score above 2 (Fig. S3 A). CELLO Prediction server also predicted SbSI-2 to be a nuclear protein (Fig. S3B). NetNES 1.1 predicted a leucine-rich nuclear export signal (NES, 131–136 aa) in SbSI-2 (Fig. S3 C, D). PROFisis Predict protein revealed that SbSI-2 has a strong protein-protein interaction domain (97–116, Fig. S4). SbSI-2 shows the possibility of phosphorylation with PKC (Protein kinase C), PKA (Protein kinase A), cdc2 and CKI (Caseine kinase I). Highest Score was 0.92 for PKC at position T-55 (Fig. S5).

### Genomic organization study

**Figure 1:**
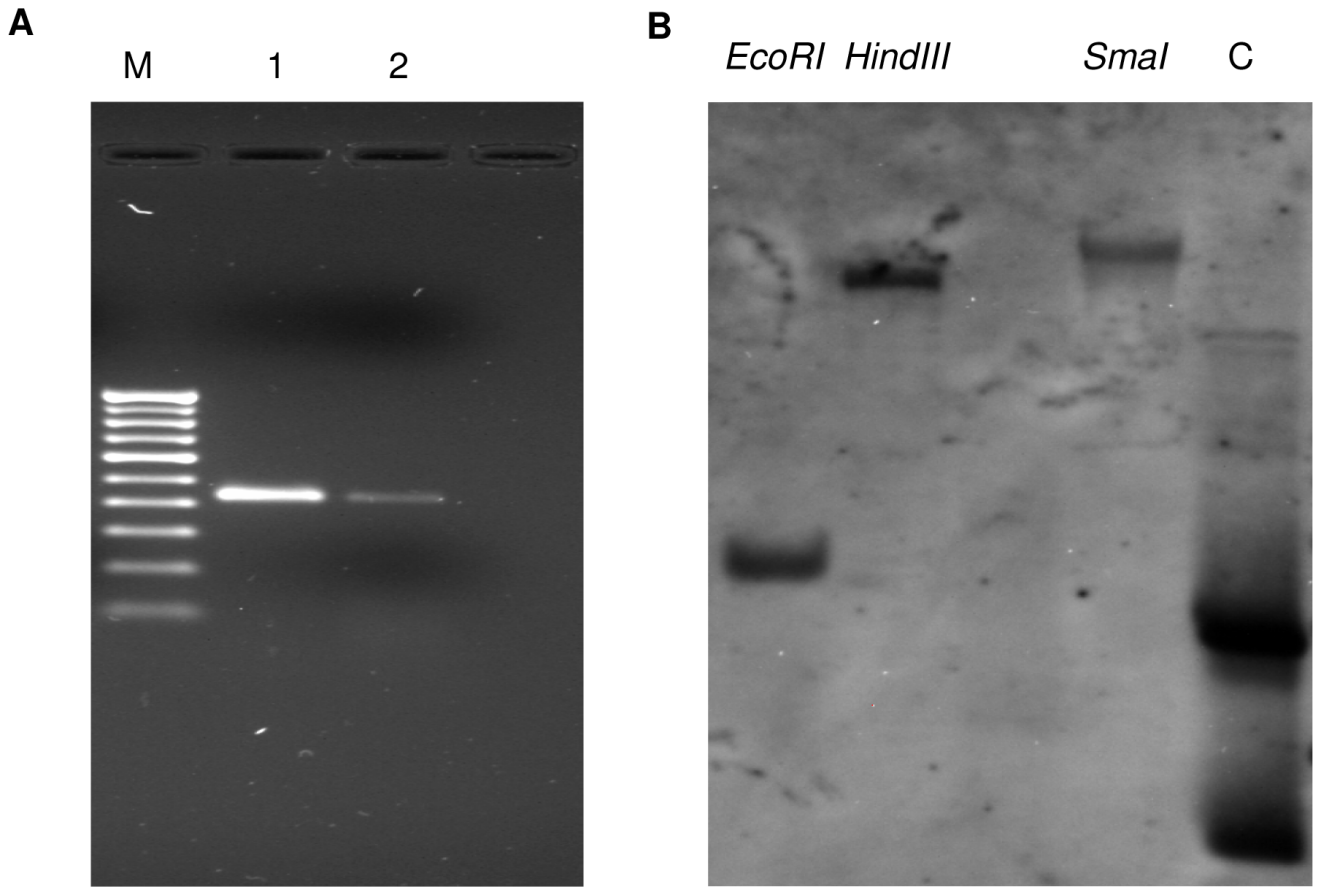
Genomic organization analysis. (A) Genomic organization study, Lane 1: Amplified *SbSI-2* PCR product from cDNA Lane 2: Amplified *SbSI-2* PCR product from genomic DNA M: 100 bp DNA ladder (B) Southern blot of *SbSI-2* gene from *Salicornia brachiata* genomic DNA. C: positive control (*SbSI-2* gene cloned in pGEMT-T Easy vector).

We have amplified *SbSI-2* ORF from genomic DNA and cDNA. Both resulting PCR products were same size on agarose gel (Fig. 1A). The amplified fragments were purified from agarose gels, cloned into a pGEM-T Easy vector and sequenced. Comparison of the genomic clone sequence with cDNA clone showed that *SbSI-2* gene has single exon structure, which is also called intronless gene.

### Copy number of *SbSI-2* gene

Southern analysis was undertaken to detect the copy number of *SbSI-2* in the *S.brachiata* genome. It was observed that *SbSI-2* probe hybridized to only single fragments of genomic DNA, digested with different restriction enzymes (*EcoRI*, *HindIII*, and *SmaI*). Southern blot revealed the presence of a single copy *SbSI-2* gene in *S. brachiata* genome (Fig. 1B).

### The SbSI-2 protein is localized in the nucleus

**Figure 2:**
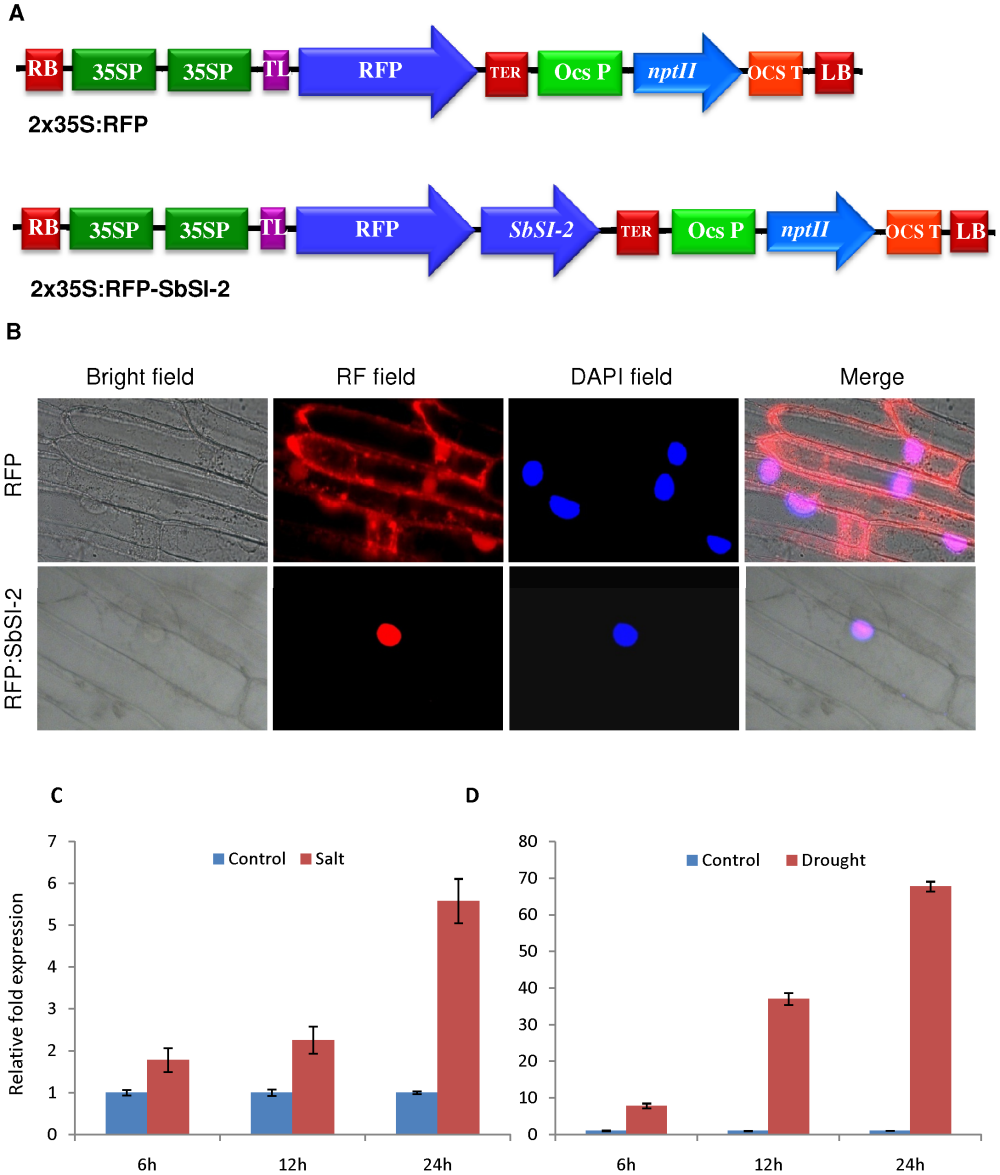
Subcellular localization of RFP:SbSI-2 fusion protein in onion epidermal cells. (A) Schematic representation of the pSITE-3CA-2X35S:RFP:SbSI-2 construct (RFP:SbSI-2) used for transient expression. (B) Cells with constructs expressing red fluorescence protein (RFP) alone and the RFP:SbSI-2 fusion protein were analyzed under bright and red fluorescence field. (C–D) Quantitative real-time PCR analysis of *SbSI-2* under salt and desiccation conditions for different time period in *S.brachiata*. The relative fold expression of *SbSI-2* at different time points under stress was calculated using the Ct value of untreated plants (control plant) at respective time points.

*In silico* sequence analysis revealed that the SbSI-2 protein has nuclear localization signals. To corroborate this we tested *in vivo* subcellular localization by transient expression assays using onion epidermal cells with pSITE-4CA constructs expressing RFP alone and the RFP:SbSI-2 fusion construct (Fig. 2A). When onion cells were transformed with RFP alone, red fluorescence signals were distributed evenly in the entire cell region, whereas in RFP:SbSI-2 fusion construct the fluorescence was accumulated in the nucleus only (Fig. 2B). These results indicate that SbSI-2 is localized in the nucleus.

### Differential expression of *SbSI-2* transcripts under salt and desiccation stresses

Expression analysis of *SbSI-2* gene was carried out by quantitative real-time PCR using cDNA from salt (NaCl) and desiccation treated plants for different time period (0, 6, 12 and 24 h). In the presence of 250 mM NaCl, *SbSI-2* transcript increased 1.5 to 4-fold (Fig. 2C) and under desiccation condition the transcript was up-regulated 2 to 70-fold (Fig. 2D). *SbSI-2* showed maximum fold change in desiccation conditions.

### Expression analysis of SbSI-2 protein in *E. coli* by SDS-PAGE

The recombinant protein was expressed by adding 1 mM IPTG at 0.6 OD_600_. The recombinant protein was induced after 2 h of treatment and reached maximum at 6 h (Fig. S6A). Presence of recombinant protein was also confirmed during liquid assay experiment after 12 hours of growth (Fig. S6B).

### Overexpression of novel *SbSI-2* in *E. coli* enhances growth during salt and osmotic stresses

**Figure 3:**
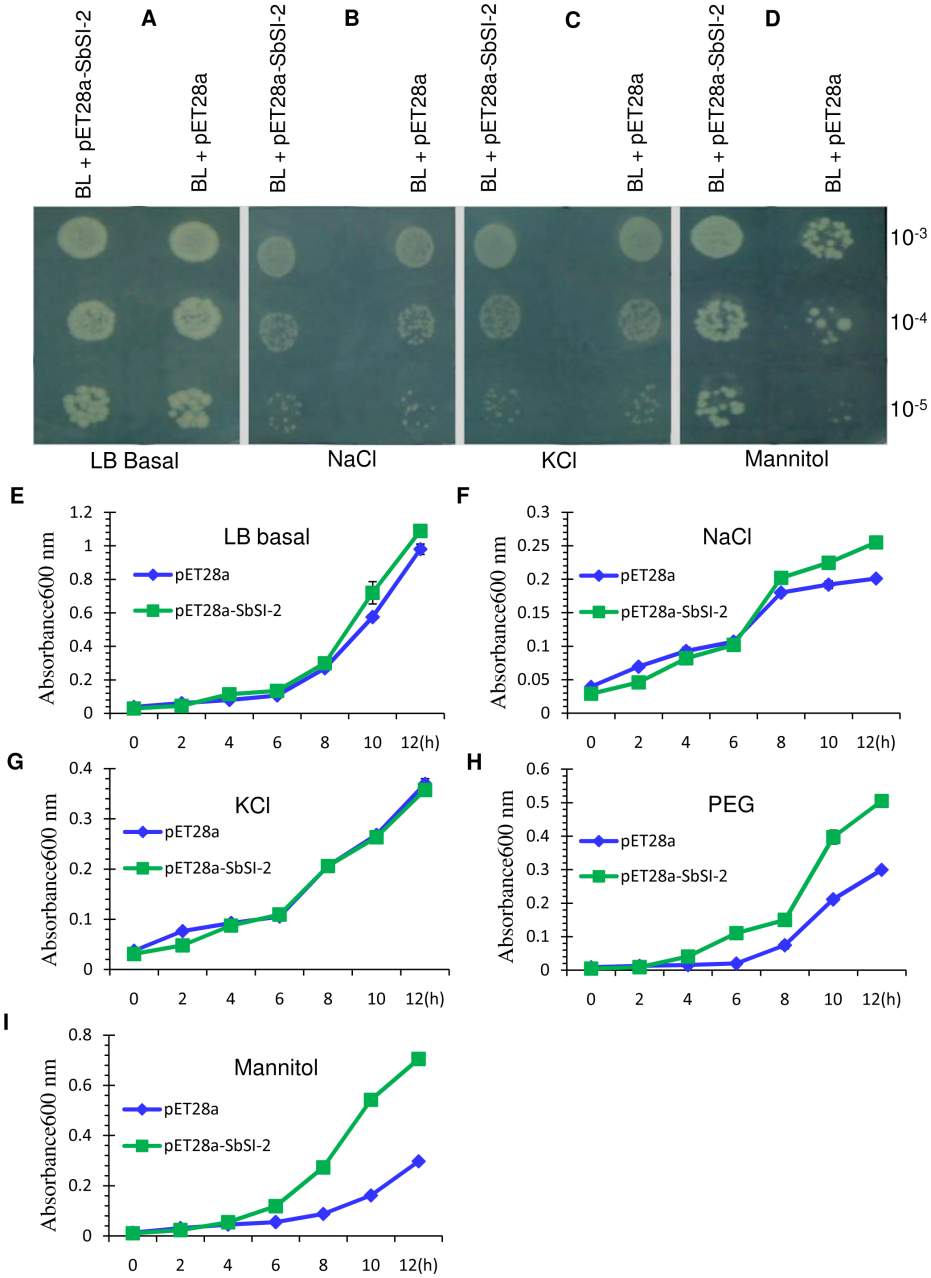
Growth analysis of recombinant *E. coli* cells having *SbSI-2*. (A–D) Spot assay of BL21 (DE3)/pET28a-SbSI-2 and BL21 (DE3)/pET28a on LB basal plates and LB supplemented with NaCl, KCl and Mannitol. Ten microliters from 10^−3^ to 10^−5^ dilutions were spotted on (A) LB basal plates, (B) LB supplemented with 500 mM NaCl, (C) 500 mM KCl, and (D) 600 mM Mannitol. (E–I) Growth analysis of novel gene *SbSI-2* was carried out in LB liquid medium with different supplements. (E) LB medium, (F) 500 mM NaCl, (G) 500 KCl, (H) 10% PEG, and (I) 600 mM Mannitol. O.D_600_ was recorded at 2 h interval up to 12 h and mean values are represented in graph.

Spot assay. pET28a-SbSI-2 recombinant cells were spotted on LB basal medium and medium supplemented with NaCl, KCl, and mannitol (Fig. 3). Recombinant (pET28a-SbSI-2) and control cells showed similar growth on LB medium in overnight grown culture (Fig. 3A). In NaCl treatment pET28a-SbSI-2 recombinant *E. coli* cells showed slightly high growth, whereas in KCl similar growth was observed compared to vector alone (Fig. 3B, C). In desiccation treatment pET28a-SbSI-2 recombinant *E. coli* cells showed significantly better growth compared to vector alone (Fig. 3D).

#### Liquid assay

Growth was also analyzed in LB liquid medium; 400 µl aliquots of pET28a-SbSI-2 recombinant and control *E. coli* BL21 (DE3) cells were inoculated in 10 ml LB liquid medium and medium supplemented with NaCl, KCl, PEG and Mannitol (Fig. 3E–I). In LB liquid medium pET28a-SbSI-2 recombinant cells and vector alone (pET28a) showed similar growth at different time points (Fig. 3E). In NaCl treatment pET28a-SbSI-2 recombinant *E. coli* cells showed higher growth 8 h after inoculation, whereas in KCl similar growth was observed compared to vector alone (Fig. 3F, G). In desiccation treatment pET28a-SbSI-2 recombinant *E. coli* cells showed better tolerance compared to vector alone (Fig. 3H, I). In the presence of 10% PEG, bacterial growth was similar up to 2 h after inoculation, but it was significantly increased in pET28a-SbSI-2 recombinant *E. coli* cells thereafter (Fig. 3H). Mannitol inhibited the growth until 4 h in both control and pET28a-SbSI-2 recombinant cells; however, after 4 h growth was significantly improved in pET28a-SbSI-2 recombinant cells compared to control cells (Fig. 3I). The liquid culture assay data showed a similar pattern of results observed with spot culture assays.

### Overexpression of *SbSI-2* enhances salinity tolerance of transgenic tobacco plants

**Figure 4:**
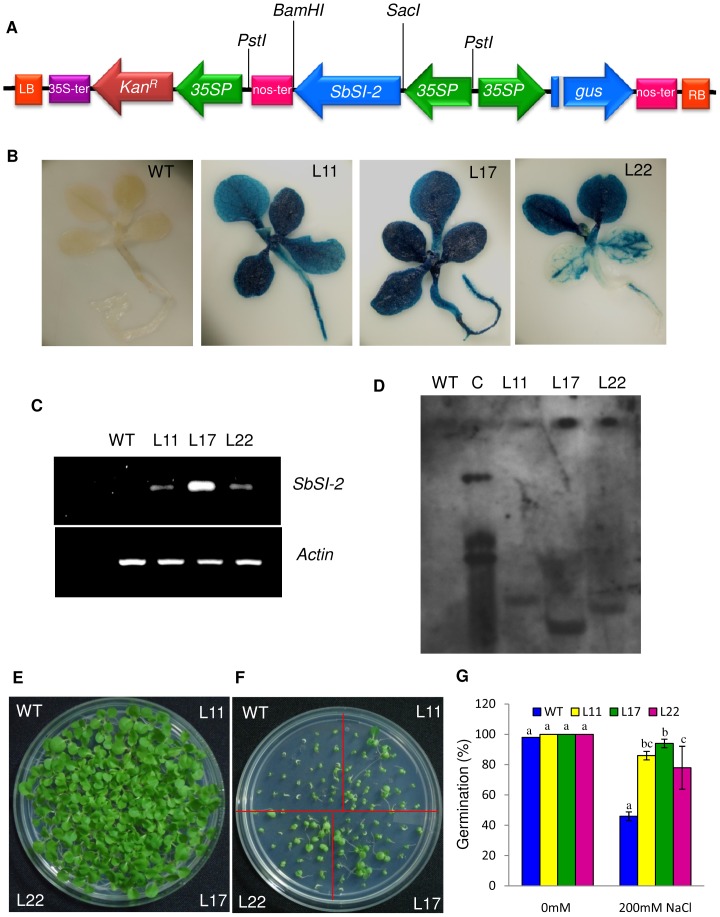
Confirmation of transgenic tobacco plants. (A) Schematic representation of the pCAMBIA2301-35S:SbSI-2 construct used to transform tobacco plants with the *SbSI-2* gene, (B) GUS assay of seedlings, showing positive GUS expression in the transgenic lines, (C) Transcript levels of the *SbSI-2* gene in transgenic lines and WT plants via semi-quantitative RT-PCR, (D) Southern analysis of transgenic lines, (E–F) Germination of seeds from transgenic lines (L11, L17 and L22) and WT plants in (E) 0 mM, and (F) 200 mM NaCl and (G) Graphs represent the percentage germination of transgenic lines (L11, L17 and L22) and WT plants in salt stress and normal condition. Mean values that were significantly different at p≤0.05 within treatment from each other are indicated by different letters (a, b and c).

The pCAMBIA2301-35S:SbSI-2 construct (Fig. 4A) was introduced into tobacco plants for *in vivo* functional characterization of *SbSI-2* gene. Putative transgenic lines were selected on kanamycin-containing medium and were subsequently verified by GUS analysis. GUS-positive transgenic lines were further verified by PCR analysis with gene specific primers and *gus*-specific primers. Thirty nine GUS and PCR positive individual transgenic lines derived from independent transgenic events were subsequently transferred to plastic pots containing garden soil and further to earthen pots after 15 days of hardening. There were no morphological differences observed between transgenic lines and WT plants under normal conditions. Seeds of 35S:SbSI-2 transgenic plants exhibited the expected 3∶1 ratio of Kan^r^/Kan^s^ during germination in kanamycin-containing medium. Three independent transgenic lines (L11, L17 and L22) were selected on the basis of GUS intensity and were further analyzed for *SbSI-2* transgene expression via semi-quantitative RT-PCR (Fig. 4B, C). The *SbSI-2*-overexpressing transgenic lines showed different levels of *SbSI-2* expression, whereas the expression of *SbSI-2* was not observed in WT plants (Fig. 4C). The L17 transgenic line exhibited maximum expression of *SbSI-2* gene (Fig. 4C). Southern blot analysis of transgenic lines L11, L17 and L22 showed single copy insertion of *SbSI-2* (Fig. 4D).

**Figure 5:**
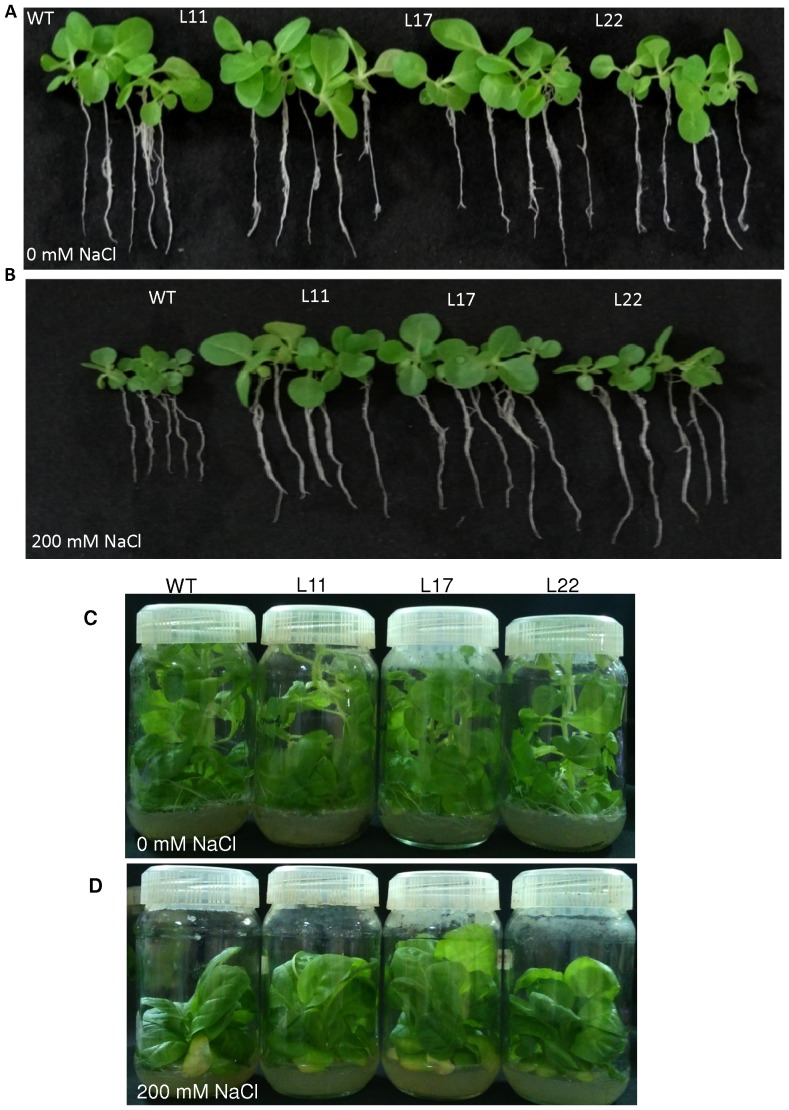
Phenotypic comparison of the growth of WT and transgenic lines overexpressing the *SbSI-2* gene under salt stress. (A–B) Growth comparison of transgenic lines (L11, L17 and L22) and WT seedlings after 30 days in (A) 0 mM, and (B) 200 mM NaCl. (C–D) Growth of whole plants from transgenic lines (L11, L17 and L22) and WT plants in (C) 0 mM and (D) 200 mM NaCl in culture bottles.

**Figure 6:**
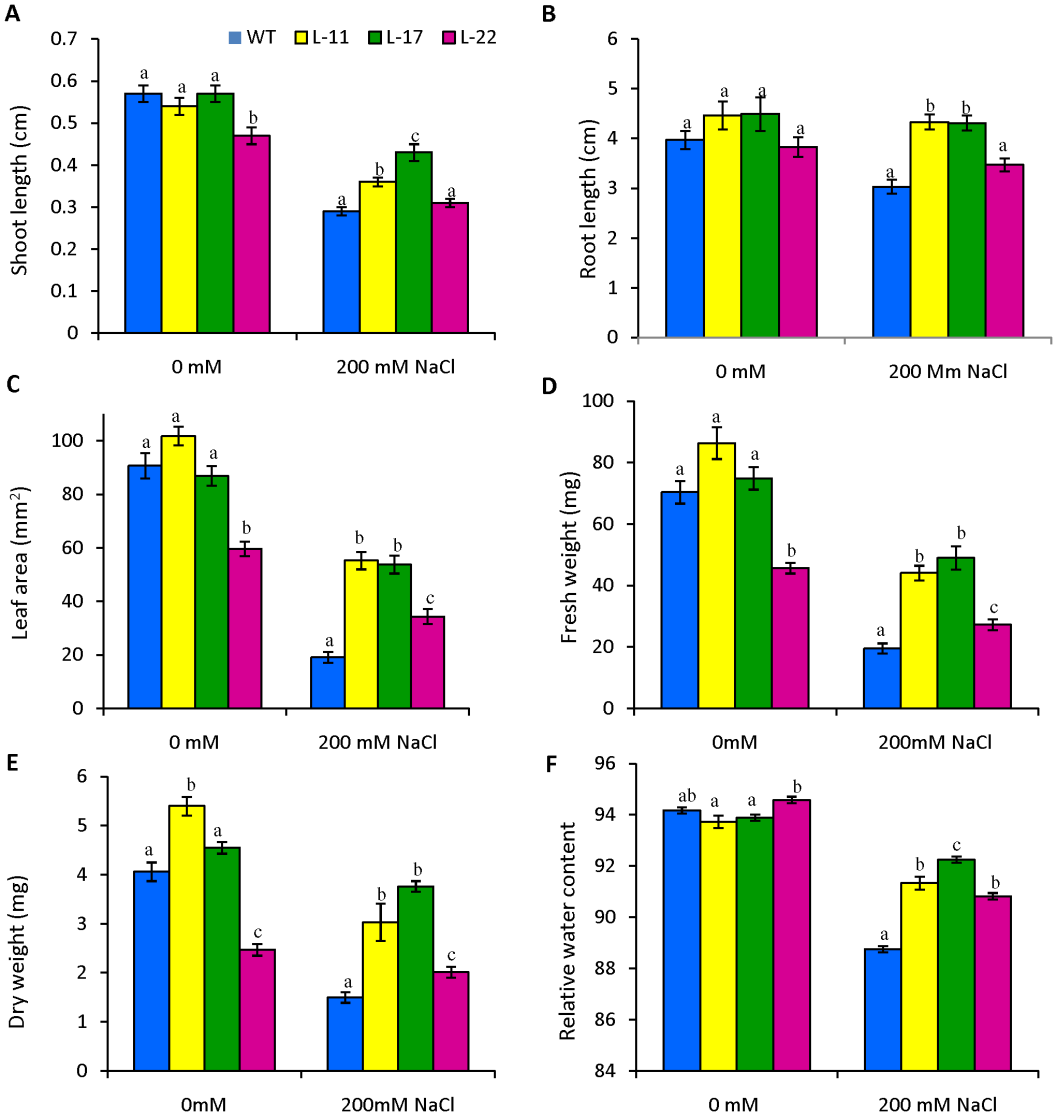
Comparison of growth parameters of seedlings from transgenic lines (L11, L17 and L22) and WT in 0 mM, and 200 mM NaCl. (A) shoot length, (B) root length, (C) leaf area, (D) fresh weight, (E) dry weight and (F) relative water content (RWC). Mean values that were significantly different at p≤0.05 within treatment from each other are indicated by different letters (a, b and c).

To study the effect of salt stress on germination, WT and transgenic lines seeds (L11, L17 and L22) were germinated in MS medium supplemented with 0 or 200 mM NaCl. Under normal conditions (0 mM NaCl) there was no difference observed between WT and transgenic seeds (Fig. 4E–G). The efficiency of germination was reduced under NaCl stress for both WT and transgenic seeds. However, transgenic seeds exhibited better germination efficiency than WT seeds under 200 mM NaCl (Fig. 4F, G). In addition to seed germination assays, the growth of transgenic seedlings exposed to salt stress condition was also examined (Fig. 5A, B). Seeds of WT and transgenic tobacco were allowed to germinate in MS medium for 8 days. Subsequently, seedlings were transferred to medium containing 0 or 200 mM NaCl. Transgenic lines of L11 and L17 exhibited significant enhancements in root and shoot length relative to WT seedlings (Fig. 6A, B). All three transgenic lines exhibited significant difference in leaf area relative to WT seedlings (Fig. 6C). Transgenic lines exhibited significant increases in fresh weight (FW), dry weight (DW) and relative water content relative to WT (Fig. 6D–F). Transgenic lines also exhibited better growth than their WT counterparts when subjected to salt stress in culture jars for long period (Fig. 5C, D).

### Overexpression of *SbSI-2* led to higher chlorophyll content, reduction in electrolyte leakage and increase in accumulation of compatible osmolytes under salt stress

**Figure 7:**
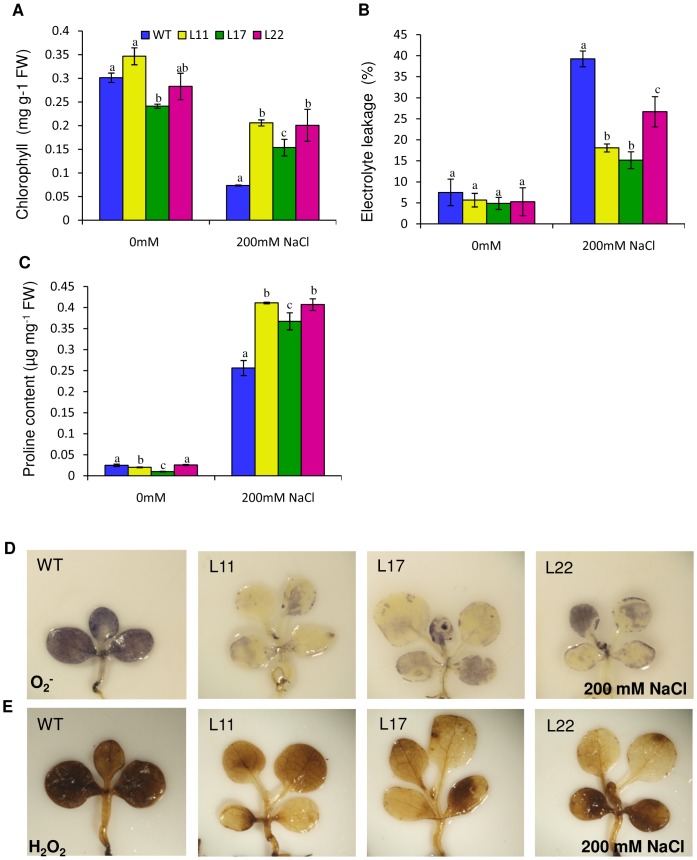
Comparison of various biochemical and physiological parameters of transgenic lines (L11, L17 and L22) and WT under salt stress. Chlorophyll (A), Electrolyte leakage (B), and proline (C) contents of transgenic (L11, L17 and L22) and WT seedlings grown in 0 mM, and 200 mM NaCl. (D–E) *In vivo* localization of O_2_^−^ and H_2_O_2_ in seedlings of 35S:SbSI-2 transgenic lines and WT under salt stress. (D) Localization of O_2_^−^ by NBT staining, (E) Localization of H_2_O_2_ by DAB staining. Mean values that were significantly different at p≤0.05 within treatment from each other are indicated by different letters (a, b and c).

The Chlorophyll content of WT and transgenic seedlings was similar under non-stress conditions and decreased upon salt stress in both WT and transgenic lines (Fig. 7A). However, the transgenic lines showed less reduction in chlorophyll content than WT seedlings (Fig. 7A). Transgenic seedlings also exhibited significantly reduced electrolyte leakage relative to WT under salt stress (Fig. 7B). Proline functions as osmoprotectent and can prevent cell dehydration and enhance stress tolerance in plants [51]. At normal condition, proline content was almost equal in WT and transgenic seedlings; however, in the presence of 200 mM NaCl, the transgenic seedlings had higher proline content relative to WT (Fig. 7C).

### Overexpression of *SbSI-2* gene reduced accumulation of reactive oxygen species (ROS) under salinity stress

WT seedlings exhibited more NBT and DAB staining than transgenic seedlings after salt stress (Fig. 7D, E). These results demonstrated that WT seedlings accumulated more O_2_^−^ and H_2_O_2_ relative to transgenic seedlings, confirming that *SbSI-2* helps to minimize oxidative stress in plants.

### Ion content analysis of *SbSI-2*-overexpressing tobacco plants under salt stress

**Figure 8:**
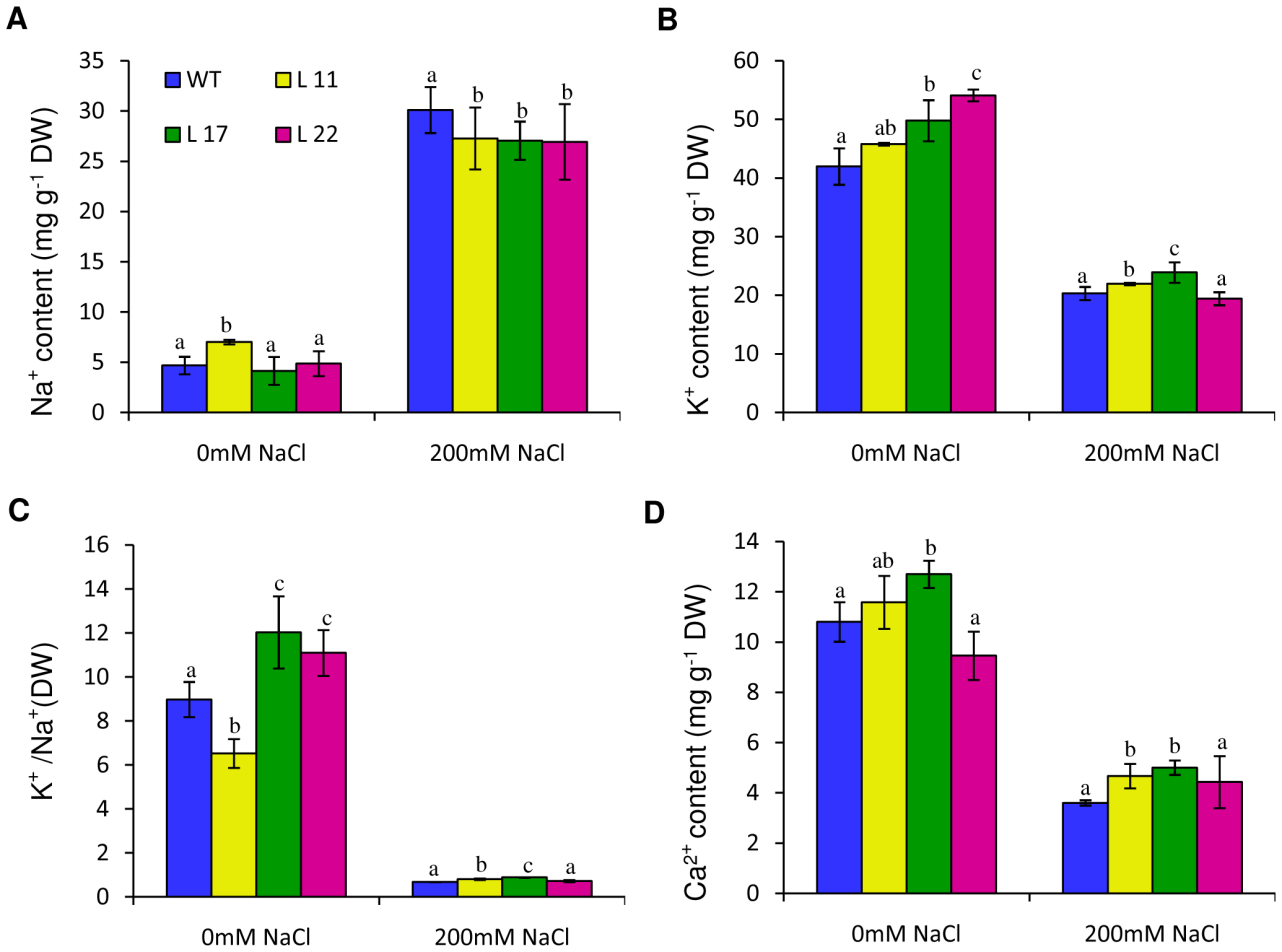
Ion content analysis. Na^+^ (A), K^+^ (B) and Ca^2+^ (D) contents in seedlings of transgenic lines (L11, L17 and L22) and WT grown in 0 mM, and 200 mM NaCl. Individual K^+^/Na^+^ ratios are shown in (C). Mean values that were significantly different at p≤0.05 within treatment from each other are indicated by different letters (a, b and c).

The Na^+^, K^+^ and Ca^2+^ content was measured in transgenic and WT seedlings grown in 0 mM and 200 mM NaCl. Under non-stress conditions transgenic and WT plants exhibited almost equal Na^+^ content (Fig. 8A). After salt stress, seedling tissues exhibited increased Na^+^ content in both transgenic lines and WT, however transgenic seedlings accumulated lower Na^+^ compared to WT seedlings (Fig. 8A). Transgenic as well as WT seedlings showed reduction in K^+^ content under NaCl stress. However, transgenic lines L11 and L17 exhibited higher K^+^ ion content relative to WT seedlings under salt stress (Fig. 8B). The L11 and L17 transgenic seedlings showed an improved K^+^/Na^+^ ratio at NaCl stress conditions relative to WT (Fig. 8C). The transgenic lines showed an increase in Ca^2+^ content as compared to WT under salt stress (Fig. 8D). It is observed that the changes in ion content are caused by NaCl both in WT and transgenic lines. However, the mode and magnitude of change are different in wild type and transgenic lines. For example, the decrease in Ca^++^ ion content in WT is 67% and that in L11, L17 and L22 is 59%, 60% and 53%, respectively (Figure 8). Therefore, the observed difference in transgenic lines vis-à-vis WT may be due to *SbSI-2*.

### *SbSI-2* expression conferred osmotic tolerance in transgenic tobacco

**Figure 9:**
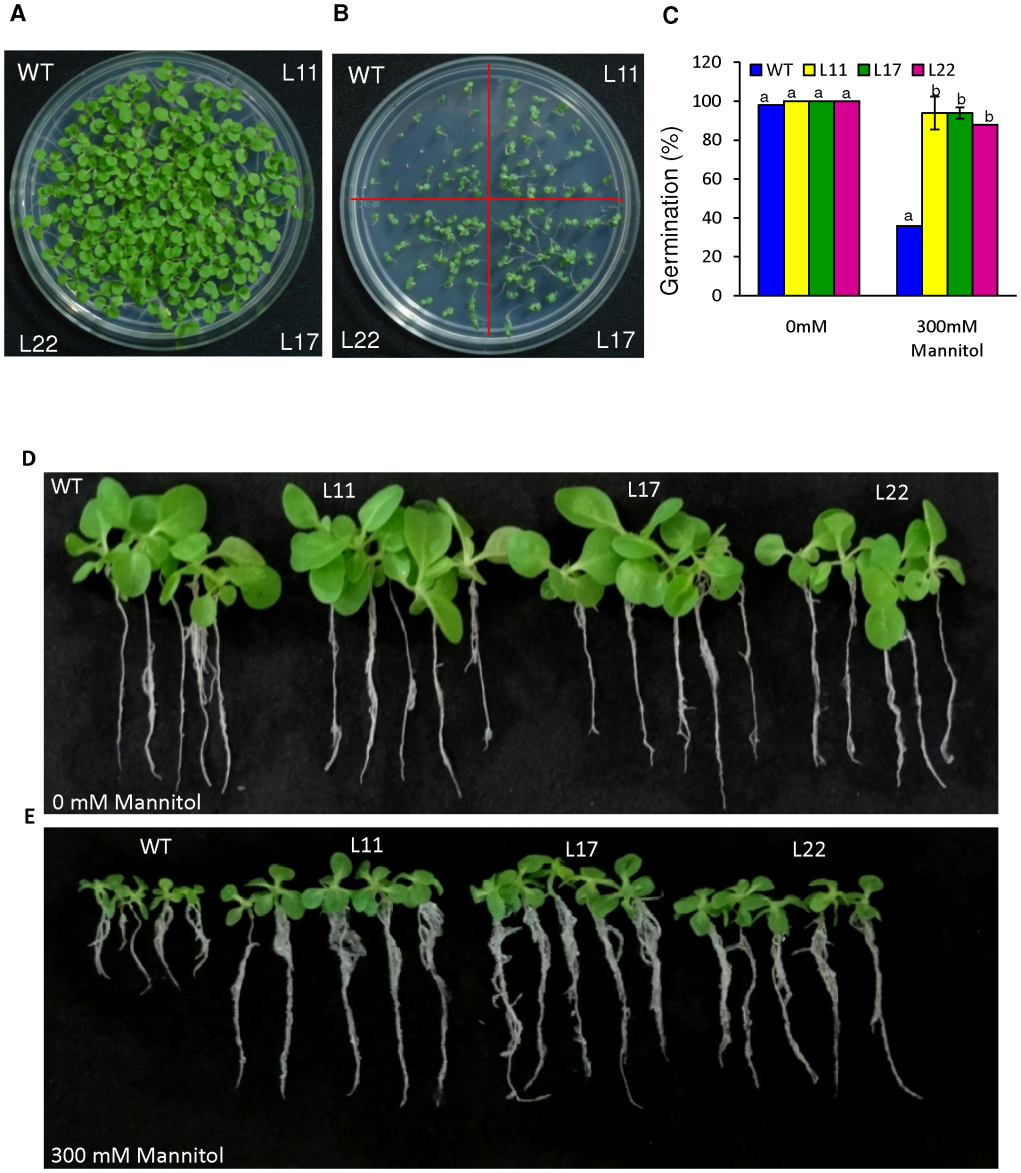
Phenotypic comparison of the growth of WT and transgenic lines overexpressing the *SbSI-2* gene under osmotic stress. (A–B) Germination of seeds from transgenic lines (L11, L17 and L22) and WT plants in (A) 0 mM, and (B) 300 mM mannitol. (C) Graphs represent the percentage germination of transgenic lines (L11, L17 and L22) and WT plants in osmotic stress (300 mM mannitol) and normal condition. (D–E) Growth comparison of transgenic lines (L11, L17 and L22) and WT seedlings in (d) 0 mM, and (e) 300 mM mannitol. Mean values that were significantly different at p≤0.05 within treatment from each other are indicated by different letters (a, b and c).

We performed osmotic tolerance tests in both WT and transgenic plants to gain a better understanding of *SbSI-2* function under dehydration. To study the effect of osmotic stress on germination, seeds of WT and transgenic lines (L11, L17 and L22) were germinated in MS medium supplemented with 0 or 300 mM mannitol. Under non-stress conditions, transgenic and WT seeds showed almost equal germination efficiency (Fig. 9A, C). At 300 mM Mannitol, transgenic seeds showed higher germination than WT seeds (Fig. 9B, C). In addition to seed germination assays, the growth of transgenic seedlings exposed to osmotic stress condition was also examined (Fig. 9D, E). Seeds of WT and transgenic tobacco were allowed to germinate in MS medium for 8 days. Subsequently, seedlings were transferred to medium containing 0, or 300 mM mannitol.

**Figure 10:**
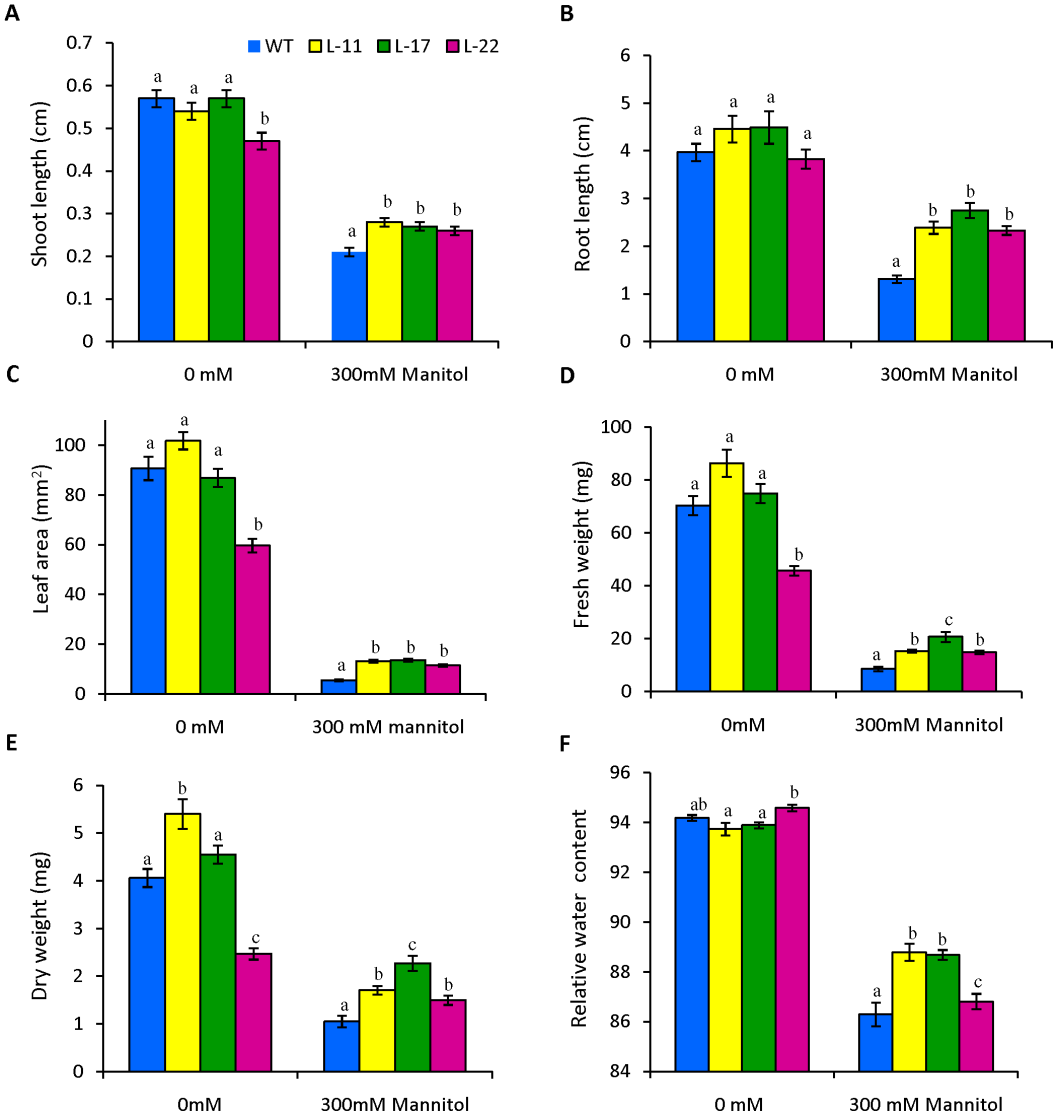
Comparison of growth parameters of seedlings from transgenic lines and WT in 0(osmotic stress). (A) shoot length, (B) root length, (C) leaf area, (D) fresh weight, (E) dry weight and (F) relative water content (RWC). Mean values that were significantly different at p≤0.05 within treatment from each other are indicated by different letters (a, b and c).

All transgenic seedlings exhibited significant difference in shoot length, root length and leaf area relative to WT under osmotic stress (Fig. 10A, B, C). Transgenic seedlings exhibited significant increases in both FW and DW relative to WT (Fig. 10D, E). WT seedlings showed signs of dehydration in the presence of mannitol stress, whereas the transgenic seedlings had better water status (Fig. 10F).

### *SbSI-2* transgenic lines showed higher chlorophyll content, lower electrolyte leakage and higher accumulation of proline in response to osmotic stress

**Figure 11:**
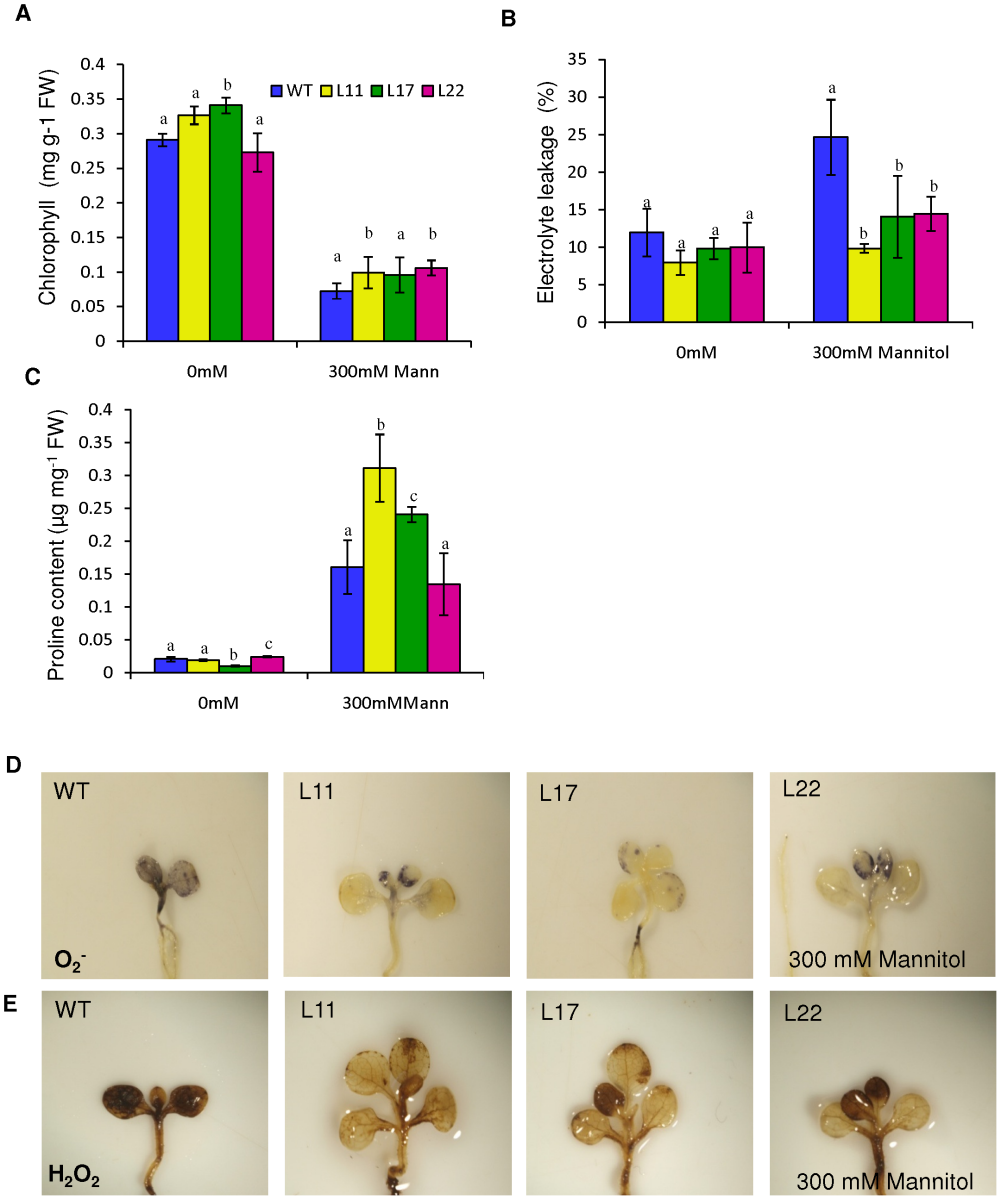
Comparison of various biochemical and physiological parameters of transgenic lines (L11, L17 and L22) and WT under osmotic stress. Chlorophyll content (A), Electrolyte leakage (B), and proline contents (C) of transgenic lines (L11, L17 and L22) and WT plants grown in the presence of 0 mM, and 300 mM mannitol. (D,E) *In vivo* localization of O_2_^−^ and H_2_O_2_ in seedlings of 35S:SbSI-2 transgenic lines and WT under osmotic stress. (D) Localization of O_2_^−^ by NBT staining, (E) Localization of H_2_O_2_ by DAB staining. Mean values that were significantly different at p≤0.05 within treatment from each other are indicated by different letters (a, b and c).

After osmotic stress, chlorophyll content reduced in both transgenic and WT seedlings compared to non-stress conditions (Fig. 11A). However, L11 and L22 showed significantly higher chlorophyll content compared to WT plants upon osmotic stress (Fig. 11A). During osmotic stress, transgenic seedlings exhibited significantly reduced electrolyte leakage as compared to WT seedlings (Fig. 11B). Proline content was also measured in osmotic stress conditions. At non-stress condition, proline content was almost similar in WT and transgenic seedlings; however, in the presence of 300 mM mannitol, L11 and L17 had higher proline content than WT (Fig. 11C).

### Overexpression of *SbSI-2* gene reduced the accumulation ROS under osmotic stress

*In vivo* localization study demonstrated that WT seedlings accumulated more O_2_^−^ and H_2_O_2_ than transgenic seedlings under osmotic stress (Fig. 11D, E).

### Expression analysis of ROS-related genes in *SbSI-2*-overexpressing transgenic tobacco plants under salinity and osmotic stress

**Figure 12:**
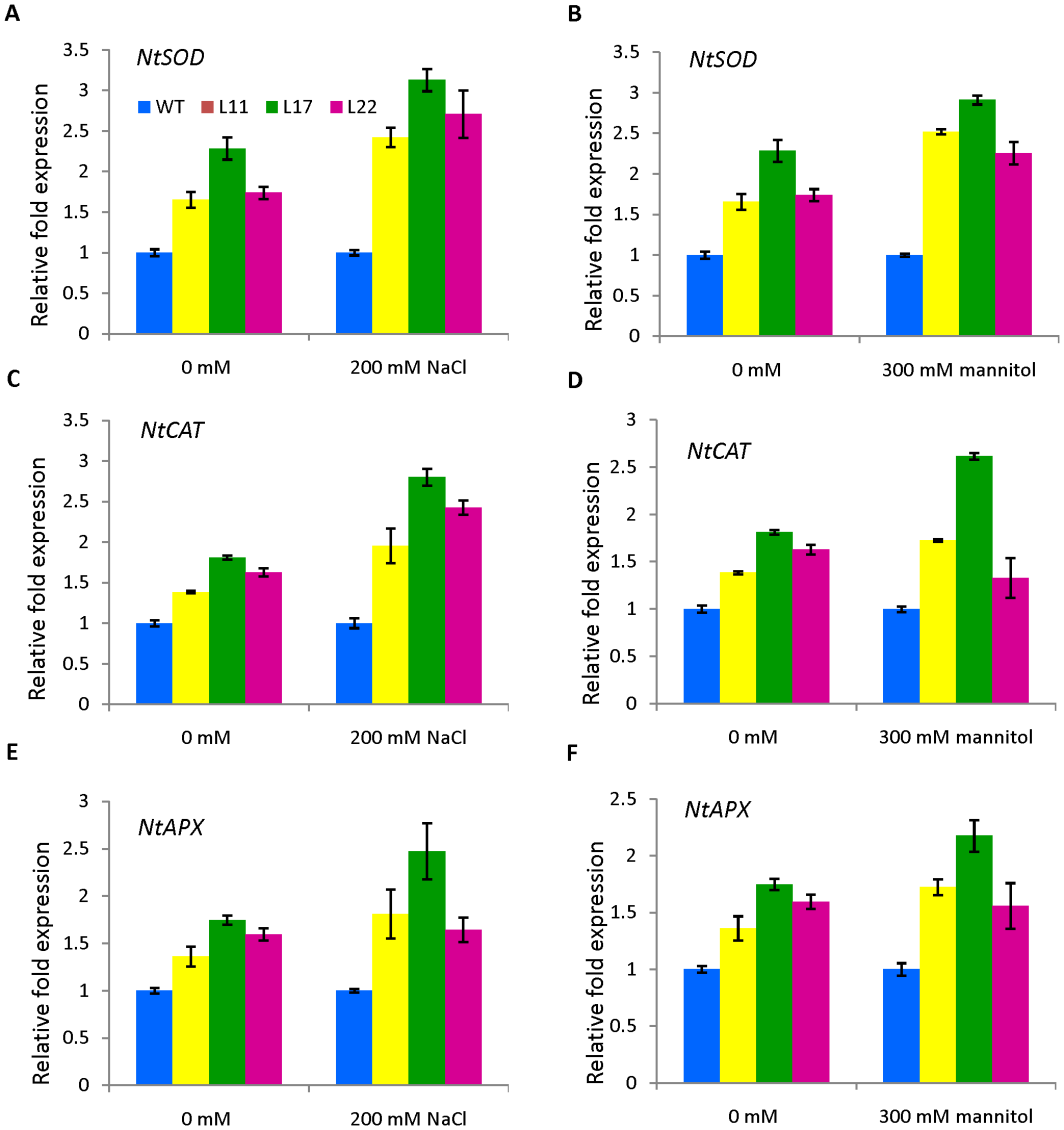
Expression analysis of ROS-related genes (*NtSOD*, *NtCAT*, *NtAPX*) in WT and *SbSI-2*-overexpressing plants by qRT-PCR. (A, C and E) Expression analysis under salt stress (200 mM NaCl) and (B, D and F) Expression analysis under osmotic stress (300 mM mannitol).

To gain further insight into molecular mechanism(s) underlying the enhanced salinity and osmotic tolerance of *SbSI-2*-overexpressing transgenic tobacco plants, we performed qRT-PCR analysis of ROS-related genes. The *NtSOD*, *NtAPX* and *NtCAT* genes encode enzymes for ROS-scavenging in plants. Transcript levels of these ROS-related genes were higher in transgenic tobacco plants as compared to WT under both salt and osmotic stress conditions (Fig. 12 A–F). Under control condition, transgenic lines also showed higher expression of ROS-related genes. These results indicate that overexpression of the *SbSI-2* gene in tobacco positively modulates expression of ROS-related genes.

### Effect of *SbSI-2* overexpression on the expression of stress-responsive TFs

**Figure 13:**
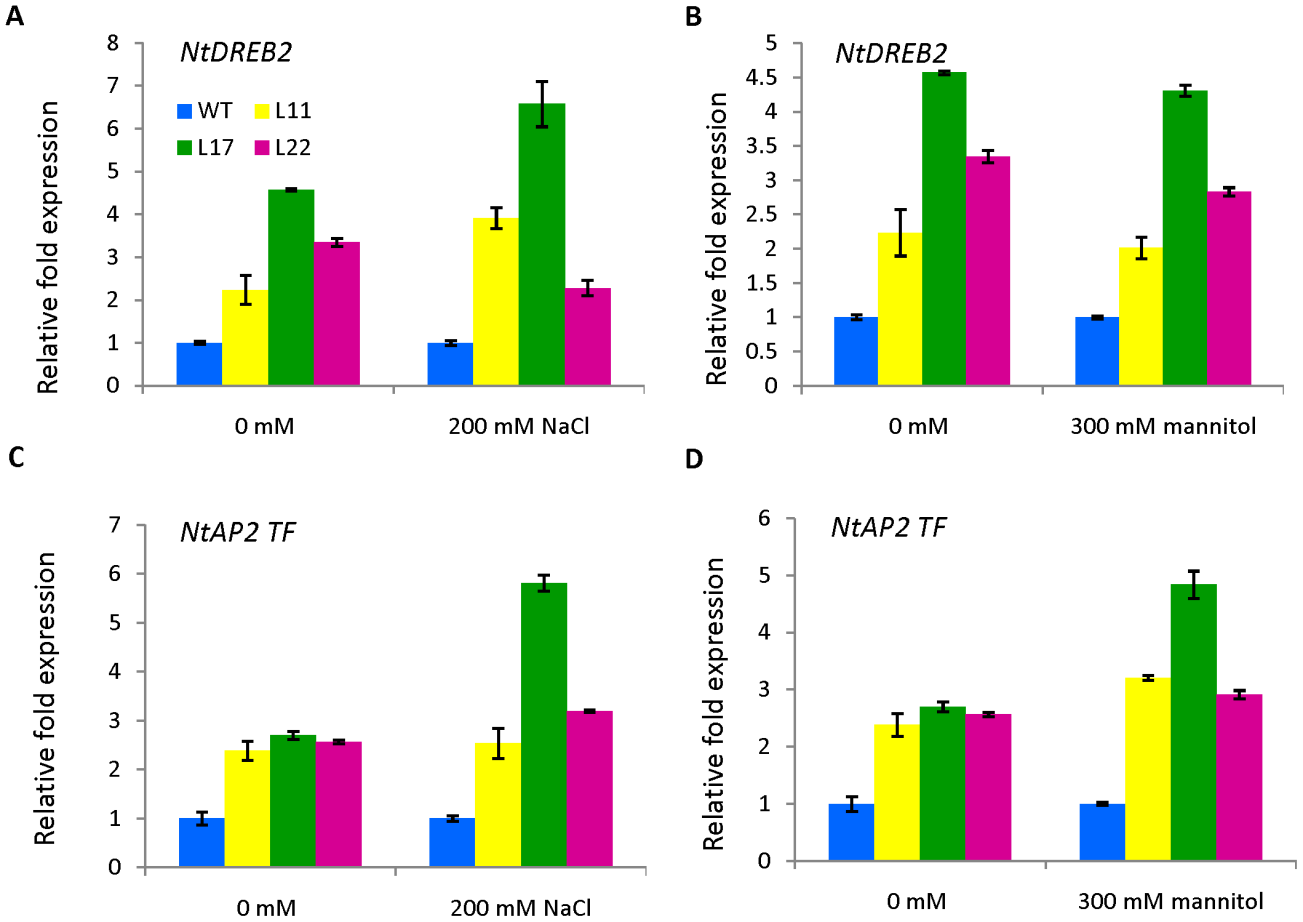
Expression analysis of stress-related transcription factors (*NtDREB2* and *AP2*-domain containing TF) in WT and *SbSI-2*-overexpressing plants by qRT-PCR. (A and C) Expression analysis under salt stress (200 mM NaCl), and (B and D) Expression analysis under osmotic stress (300 mM mannitol).

To investigate how *SbSI-2* increases salt and osmotic stress tolerance, the expression levels of some stress-responsive TFs (*NtDREB2* and *AP2*-domains containing TF) were evaluated in transgenic and WT plants. Expression levels of *NtDREB2* and *AP2*-domains containing TF were higher in transgenic tobacco plants as compared to WT under both stress and control conditions (Fig. 13). However relative fold increase of transcript (NtDREB2) is seen only in L11 and L17 under salt stress (Figure 13A), but no relative fold increase is observed under osmotic stress relative to control condition (Figure 13B). The NtAP2 transcription factor gene showed relative fold increase in transcript level only in L17 and L22 line under salt stress relative to control condition (Figure 13C). In case of osmotic stress, all transgenic lines showed increase in transcript level of NtAP2 TF (Figure 13D). On the basis of these results, we speculate that over-expression of *SbSI-2* positively modulates expression of stress responsive TFs.

## Discussion

Abiotic stress reduces plant growth and survival. Plants survive various stresses by controlling responses at both cellular and molecular level. Plant adaptation to abiotic stress involves a plethora of genes related to ion homeostasis, compatible osmolytes synthesis, ROS-scavenging and antioxidant defence mechanism. Characterization of unknown genes is an important and challenging task in deciphering their role in stress tolerance. In our previous study we have identified 270 unknown/hypothetical genes and 12 miRNAs [25], [52]. Out of 270 unknown/hypothetical genes 90 unknown genes confirmed their role in salt stress by reverse Northern analysis [25]. The *SbSI-2* cDNA fragment spanning the entire open reading frame (ORF) was cloned and sequenced (Gen-Bank accession number JX872273). Amino acid sequence analysis of SbSI-2 by different *in silico* tools revealed important features like three distinct regions, nuclear localization signals (NLS), leucine-rich nuclear export signals (NES), strong protein-protein interaction domain and phosphorylation sites (Figs. S2, S3, S4, S5). Transient expression of the RFP:SbSI2 fusion protein also showed that SbSI-2 is a nuclear-localized protein (Fig. 2B), suggesting that SbSI-2 may function in the nucleus. Nuclear localization is an integral part of abiotic stress response. Many stress-associated proteins, like some TFs (bZIP, DREB, MYC/MYB, NAC, C_2_H_2_ zinc finger protein Msn2P), some kinase gene and a number of functional proteins belong to a subset of cellular proteins that localize to the nucleus [11], [12], [53], [54], [55], [56], [57], [58], [59]. Genomic organization study showed that *SbSI-2* is an intronless gene (Fig. 1A). Genes without introns are a characteristic feature of prokaryotes, but there are still a number of intronless genes in eukaryotes. Study these eukaryotic genes that have prokaryotic architecture could help to understand the evolutionary patterns of related genes and genomes [60]. Intronless genes are good candidate pool for subsequent functional and evolutionary analysis. It has been also reported that many intronless genes remained conserved in archaea, bacteria, fungi, plants, metazoans, and other eukaryotes during evolutions [60]. Southern blot analysis revealed the presence of a single copy of *SbSI-2* in the *S. brachiata* genome (Fig. 1B).

*SbSI-2* showed the increased mRNA expression by salt stress and desiccation, signifying that *SbSI-2* plays an important role in abiotic stress tolerance. Heterologous expression of *SbSI-2* in *E. coli* cells demonstrated that pET28a-SbSI-2 recombinant *E. coli* cells showed better tolerance to desiccation and salinity compared to vector alone (Fig. 3). Similar to our study, a few earlier publications have also reported better growth of *E. coli* cells by overexpression of other plant stress-associated genes [30], [34], [61], [62], [63], [64], [65]. PM2, a group 3 LEA protein from soybean, conferred salt stress tolerance in *E. coli* [61]. Expression of phytochelatin synthase in *E. coli* resulted in better protection to heat, salt, carbofuran (pesticide), cadmium, copper and UV stress [61]. It has been reported that the SbDREB2A transcription factor conferred tolerance to different stress conditions in *E. coli*. Yadav et al. [34] reported that a novel salt-inducible gene *SbSI-1* confers salt and desiccation tolerance in *E. coli*. Recently, Jin-long et al. [65] showed that the expressed novel dirigent protein ScDir from sugarcane had increased *E. coli* tolerance to PEG and NaCl. To further understand the function of *SbSI-2* under abiotic stress, we developed *SbSI-2*-overexpressing transgenic tobacco plants. Testing for a range of physiological parameters it was found that tobacco plants overexpressing *SbSI-2* have improved salt and osmotic tolerance, accompanied by better growth, higher seed germination, better water status, and higher photosynthetic rate as compared to WT plants (Figs. 4; 5; 6; 7A; 9; 10; 11A). The better water status in transgenic plants, indicate that SbSI-2 helps in water retention during NaCl and osmotic stress.

The relative abundance of proline and total soluble sugar are important biochemical indicators of salinity and drought stress in plants [66]. It has been reported that increased proline content under various environmental stresses significantly improved plant stress tolerance [67], [68], [69]. Proline protects the plants in response to salt and drought stresses by osmoprotection and ROS-scavenging, which contributes to membrane stability and mitigates the disruptive effect of stress [70]. Transgenic plants overexpressing *SbSI-2* accumulate higher proline relative to WT plants during salt and osmotic stress (Figs. 7C; 11C).

In the present study, transgenic plants facing salt and osmotic stress exhibited low electrolyte leakage (Figs. 7B; 11B). Plants experiencing abiotic stress often exhibit symptoms of oxidative stress as evidenced by elevated accumulation of ROS and MDA contents [71]. It has been reported that plants maintain their ROS pools at low levels in order to minimize cellular damage caused by oxidative stress [72], [73]. ROS accumulation depends on the balance between production and contemporaneous scavenging [74]. Plant cells have a complex antioxidant defence system for ROS-detoxification [75]. The overexpression of *SbSI-2* reduced accumulation of ROS in response to salt and osmotic stresses, which indicates a reduced oxidative damage resulting from stress (Figs. 7D, E; 11D, E).

Salinity stress leads to increase in cellular Na^+^ and decrease in K^+^, which causes an ion-toxic effect in cells, physiological drought, and lack of nutrients [76]. Therefore, maintenance of a low intracellular Na^+^ concentration or a high cytosolic K^+^/Na^+^ ratio is crucial for salt tolerance in plants [77], [78]. Studies have shown that, among glycophytes such as wheat, Na^+^ efflux and high K^+^/Na^+^ ratio are the key mechanisms involved in salt tolerance [79], [80]. To decipher the mechanism by which *SbSI-2* overexpression improves salt tolerance, ion contents analysis were carried out in transgenic and WT plants under non-stress and salt-stress conditions. *SbSI-2*-overexpressing transgenic tobacco seedlings accumulated lower Na^+^ and higher K^+^ content after salt stress, with improved K^+^/Na^+^ ratio (Fig. 8), suggesting that *SbSI-2* overexpression ensures better physiological activities and imparts salt tolerance by increasing the K^+^/Na^+^ ratio. On the basis of these physiological and biochemical analysis, we can speculate that improved salt and osmotic tolerance in the *SbSI-2*-overexpressing transgenic lines is correlated with high water retention capacity, higher accumulation of osmolytes, high K^+^/Na^+^ ratio, reduced electrolyte leakage and less accumulation of ROS.

To gain further insight into enhanced abiotic stress tolerance in *SbSI-2*-overexpressing transgenic tobacco at the molecular level, the expression levels of ROS-scavenging genes (*NtSOD*, *NtAPX* and *NtCAT*) and some stress-associated TFs (*NtDREB2* and *AP2*-domains containing TF) were evaluated in transgenic and WT plants. *SbSI-2*-overexpressing lines showed higher expression of genes encoding ROS-scavenging enzymes (SOD, APX and CAT) under salt or osmotic stress, which was consistent with the lower levels of ROS in transgenic seedlings relative to WT seedlings (Fig. 12). In present study, the bioinformatics analysis and transient expression assays of the RFP:SbSI2 fusion protein showed that SbSI-2 is a nuclear-localized protein and has a strong protein-protein interaction domain which possibly interact with transcription factors that regulate the expression of the abiotic stress-responsive genes. To further investigate, we have carried out expression analysis of two stress-associated TFs *DREB2* and *AP2*-domains containing TF in transgenic lines and WT plants. *SbSI-2*-overexpressing plants showed up-regulated expression of *DREB2* and *AP2*-domains containing TF, which in turn enhanced the expression of abiotic stress-responsive genes. These results demonstrate that *SbSI-2* might play vital positive regulatory role in abiotic stress tolerance. Taken together, we propose that the improved salt and osmotic tolerance in *SbSI-2*-overexpressing transgenic plants might be achieved by elevated expression of stress-associated TFs, which in turn up-regulate the expression of abiotic stress-responsive genes. However, further study is needed to confirm this.

In conclusion, we have cloned and characterized a novel salt-inducible gene *SbSI-2* from the extreme halophyte *Salicornia brachiata*. The *SbSI-2* gene showed up-regulation by different abiotic stresses. Subcellular localization study indicated that the SbSI-2 protein is nuclear-localized. The *SbSI-2* gene was transformed in *E. coli* and tobacco for functional characterization. pET28a-SbSI-2 recombinant *E. coli* cells showed higher tolerance to desiccation and salinity compared to vector alone. Further, overexpression of the *SbSI-2* gene in tobacco conferred salt- and osmotic tolerance by promoted seed germination, improved growth parameters, higher relative water content, higher K^+^/Na^+^ ratio, higher chlorophyll and elevated accumulation of of compatible osmolytes as compared to control plants. Transgenic plants exhibited reduction in electrolyte leakage and ROS in response to salt and osmotic stresses. Overexpression of *SbSI-2* also enhanced the transcript levels of ROS-scavenging genes and some stress-responsive TFs under salt and osmotic stresses. The present study demonstrates that *SbSI-2* might play an important positive modulation role in abiotic stress tolerance and suggests that it could be a potential bioresource for bioengineering abiotic stress tolerance in crop plants.

## Supporting Information

Figure S1 **Nucleotide sequence with deduced amino acid sequence and predicted secondary structure of SbSI-2 protein.** (A) Full-length cDNA and deduced amino acid sequence of *SbSI-2*. Start codon (ATG) and stop codon (TGA) are indicated by green and red colour, respectively. The 5′ and 3′-UTR regions are indicated by blue color. (B) Predicted secondary structure viz. helix, strands and coils as indicated by pink rods, arrow and solid lines, respectively.
(PDF)

Figure S2 **ScanProsite results together with ProRule-based predicted intra-domain features.**
(PDF)

Figure S3 ***In silico*** **localization analysis by various bioinformatics softwares.** (A) Nuclear localization signals and their positions by the WoLF PSORT. (B) Cello prediction result for localization. (C) and (D) Leucine-rich nuclear export signals (NES) prediction by NetNES 1.1 server using a combination of neural networks and hidden Markov models. The prediction server calculates NES score from Hidden Markov Models (HMMs) and Artificial Neural Network (ANN) scores (all three values are given for each residue).
(PDF)

Figure S4 **Protein-protein binding domain detected by PROFisis PredictProtein server.** Blue underlined text shows the strong protein-protein interaction domain.
(PDF)

Figure S5 **Predicted Phosphorylation sites in SbSI-2 protein by NetPhosK 1.0 software.**
(PDF)

Figure S6 **SDS-PAGE analysis of expression of recombinant protein (shown by arrow) in** ***E. coli*** **BL21 (DE3) cells expressing** ***SbSI-2*** **gene.** (A) M marker (kDa), Lane 1: uninduced protein, Lane 2: induced protein 2 h, Lane 3: induced protein 4 h, Lane 4: induced protein 6 h. (B) M marker (kDa), Lane 1 and 2: induced protein 6 h, Lane 3 and 4: uninduced protein, Lane 5: induced protein in liquid assay after 12 h of growth.
(PDF)
