# Supplementary material for: A Novel Gene SbSI-2 Encoding Nuclear Protein from a Halophyte Confers Abiotic Stress Tolerance in E. coli and Tobacco
Source: PLoS One. 2014 Jul 7;9(7):e101926. doi: 10.1371/journal.pone.0101926 (PMC4084957; doi:10.1371/journal.pone.0101926)

Figure S1

A

1 – GCAAACCACAGCCTTTTTCCCTTTAC – 26
27 – ATGGGATTTCATTCCTTTGACGTCTTCTACTTTTTCTTTCTTTCTTGCCCTAACCCTAAT – 86
1 – M G F H S F D V F Y F F F L S C P N P N – 20

87 – TTCTGTTTTTCTCTCTCTTTATCAAATTTCCAGAAAAAATCTCGAGATAAAGAGAGGGAG – 146
21 – F C F S L S L S N F Q K K S R D K E R E – 40

147 – ATTCAGACCAATTATCCTACATTCAAAAAATCCAAAAAAAAATACAAAACCCAGAAAGAAA – 206
41 – I Q T N Y P T F K K S K K N T K P R K K – 60

207 – AAGGCAAGAAAAAAAAATGGGGAAGTATTCAGAGTTGATAGATGCAGGGGTAAGAATTGCT – 266
61 – K A R K K M G K Y S E L I D A G V R I A – 80

267 – GCCCGATTTCATTCTCACTGCCCGCAAACTGCCCGGATGTATTATCACCCTCCTCCTCCG – 326
81 – A R F H S H C P Q T A R M Y Y H P P P P – 100

327 – ACCACCGCGGAGTCGGGCCCCACCCAACGATATCCACCGCAAGACGGCGGCGTTTTGGGT – 386
101 – T T A E S G P T Q R Y P P Q D G G V L G – 120

387 – TGCAAAGGAAGCTCTTCTGGAGTTGATATTACCAAGGATCTGATTCTTCATTCGATTTGT – 446
121 – C K G S S S G V D I T K D L I L H S I C – 140

447 – TGA – 449
141 – *
450 – TTTTTAAAAAAAAAATTCTGATTGGAAGCTAGAGTTATGTTAGAAACATTTAGGT
TGAATCCTATTAACACAAAAAAAAAAAAAAAAAAA – 537

B

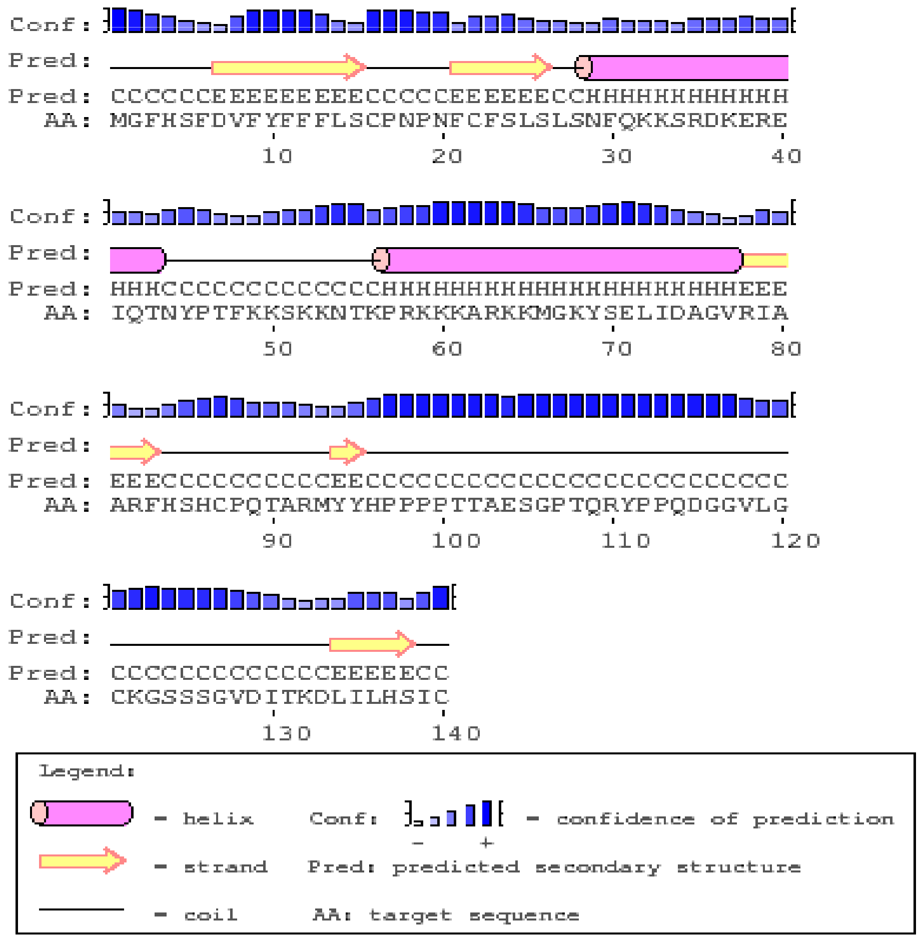

Supplement: Figure S1 — Nucleotide sequence with deduced amino acid sequence and predicted secondary structure of SbSI-2 protein. (A) Full-length cDNA and deduced amino acid sequence of SbSI-2. Start codon (ATG) and stop codon (TGA) are indicated by green and red colour, respectively. The 5′ and 3′-UTR regions are indicated by blue color. (B) Predicted secondary structure viz. helix, strands and coils as indicated by pink rods, arrow and solid lines, respectively. (PDF) [file pone.0101926.s001.pdf]
