# Supplementary material for: A Novel Gene SbSI-2 Encoding Nuclear Protein from a Halophyte Confers Abiotic Stress Tolerance in E. coli and Tobacco
Source: PLoS One. 2014 Jul 7;9(7):e101926. doi: 10.1371/journal.pone.0101926 (PMC4084957; doi:10.1371/journal.pone.0101926)
Supplement: Figure S2 — ScanProsite results together with ProRule-based predicted intra-domain features. (PDF) [file pone.0101926.s002.pdf]

Figure S2

This view shows ScanProsite results together with ProRule-based predicted intra-domain features.

Hits by patterns: [3 hits (by 3 distinct patterns) on 1 sequence]

USERSEQ1

(140 aa) 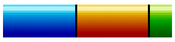

USERPAT1 :
Pattern (1 - 60): MGFHSFDVFYFFFLSCPNPNFCFSLSLSNFQKKSRDKEREIQTN YPTFKKSKKNTKPRKK

USERPAT2 :
Pattern (61 -120): KARKKMGKYSELIDAGVRIAARFHSHCPQTARMYYHPPPPT TAESG PTQRYPPDGGVLG

USERPAT3 :
Pattern (121 - 140): CKGSSSGVDITKDLILHSIC
