# Supplementary material for: A Novel Gene SbSI-2 Encoding Nuclear Protein from a Halophyte Confers Abiotic Stress Tolerance in E. coli and Tobacco
Source: PLoS One. 2014 Jul 7;9(7):e101926. doi: 10.1371/journal.pone.0101926 (PMC4084957; doi:10.1371/journal.pone.0101926)
Supplement: Figure S3 — In silico localization analysis by various bioinformatics softwares. (A) Nuclear localization signals and their positions by the WoLF PSORT. (B) Cello prediction result for localization. (C) and (D) Leucine-rich nuclear export signals (NES) prediction by NetNES 1.1 server using a combination of neural networks and hidden Markov models. The prediction server calculates NES score from Hidden Markov Models (HMMs) and Artificial Neural Network (ANN) scores (all three values are given for each residue). (PDF) [file pone.0101926.s003.pdf]

Figure S3

A

NUCDISC: discrimination of nuclear localization signals
pat4: KPRK (4) at 56
pat4: PRKK (4) at 57
pat4: RKKK (5) at 58
pat7: PTFKKSK (3) at 46
pat7: PRKKKAR (5) at 57
bipartite: KKSKKNTKPRKKKARKK at 49
bipartite: KKNTKPRKKKARKKMGK at 52
content of basic residues: 17.1%
NLS Score: 2.18

B

CELLO Prediction:

Nuclear	2.075*
Mitochondria	1.334
Extracellular	0.559
Cytoplasmic	0.440
Chloroplast	0.283
PlasmaMembrane	0.158
Peroxisomal	0.037
Lysosomal	0.028
Vacuole	0.027
ER	0.025
Cytoskeletal	0.024
Golgi	0.011

C

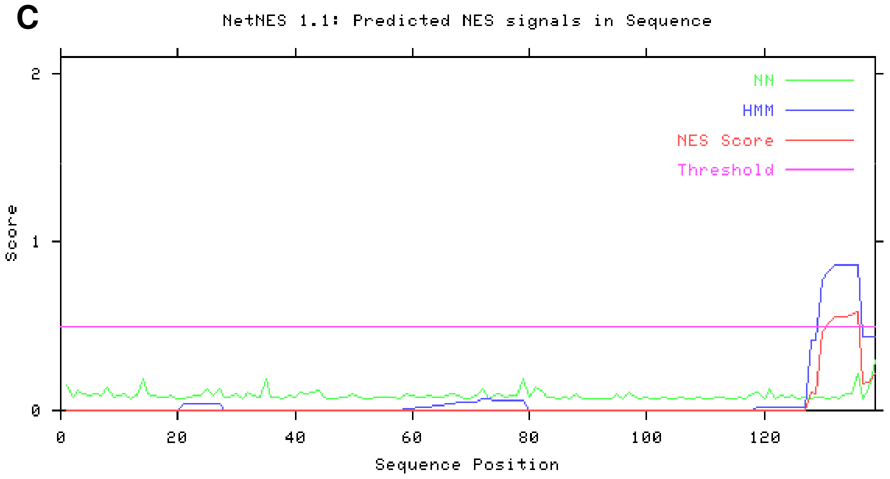

D

>Sequence - NetNES 1.1 prediction

Seq-Pos-Residue	ANN	HMM	NES	Predicted
Sequence-131-T	0.073	0.827	0.517	Yes
Sequence-132-K	0.080	0.862	0.553	Yes
Sequence-133-D	0.071	0.862	0.552	Yes
Sequence-134-L	0.095	0.862	0.557	Yes
Sequence-135-I	0.102	0.862	0.561	Yes
Sequence-136-L	0.214	0.861	0.588	Yes
