# Supplementary material for: A Novel Gene SbSI-2 Encoding Nuclear Protein from a Halophyte Confers Abiotic Stress Tolerance in E. coli and Tobacco
Source: PLoS One. 2014 Jul 7;9(7):e101926. doi: 10.1371/journal.pone.0101926 (PMC4084957; doi:10.1371/journal.pone.0101926)
Supplement: Figure S4 — Protein-protein binding domain detected by PROFisis PredictProtein server. Blue underlined text shows the strong protein-protein interaction domain. (PDF) [file pone.0101926.s004.pdf]

Figure S4

Protein-Protein binding domain

```
>query: strech=5 crowd_predictions=7 gap=20 itr=0
MGFHSFDVFYFFFLSCPNPNFCFSLSLSNFQKKSRDKERE
PP-PP-----P--------P--------------------

IQTNYPTFKKSKKNTKPRKKKARKKMGKYSELIDAGVRIA
----PP----------------P-----------------

ARFHSHCPQTARMYYHPPPPTTAESGPTQRYPPQDGGVLG
---P-------P-----PPPPPPPPPPPPPPPPPPP--PP

CKGSSSGVDITKDLILHSIC
PPP-P--------------P
```
