# Supplementary material for: A Novel Gene SbSI-2 Encoding Nuclear Protein from a Halophyte Confers Abiotic Stress Tolerance in E. coli and Tobacco
Source: PLoS One. 2014 Jul 7;9(7):e101926. doi: 10.1371/journal.pone.0101926 (PMC4084957; doi:10.1371/journal.pone.0101926)
Supplement: Figure S5 — Predicted Phosphorylation sites in SbSI-2 protein by NetPhosK 1.0 software. (PDF) [file pone.0101926.s005.pdf]

Figure S5

Method: NetPhosK without ESS filtering:
Query: Sequence

Site	Kinase	Score
S-15	PKA	0.50
S-28	PKC	0.79
T-47	PKC	0.91
S-51	PKC	0.86
S-51	PKG	0.63
T-55	PKC	0.92
S-70	PKA	0.52
S-85	RSK	0.52
S-85	PKA	0.72
T-90	PKC	0.69
T-108	PKC	0.72
S-124	CKI	0.53
S-125	PKA	0.59
S-125	cdc2	0.52

Highest Score: 0.92 PKC at position 55
