# Supplementary figures and images for: A Novel Gene SbSI-2 Encoding Nuclear Protein from a Halophyte Confers Abiotic Stress Tolerance in E. coli and Tobacco
Source: PLoS One. 2014 Jul 7;9(7):e101926. doi: 10.1371/journal.pone.0101926 (PMC4084957; doi:10.1371/journal.pone.0101926)

**Figure S6**

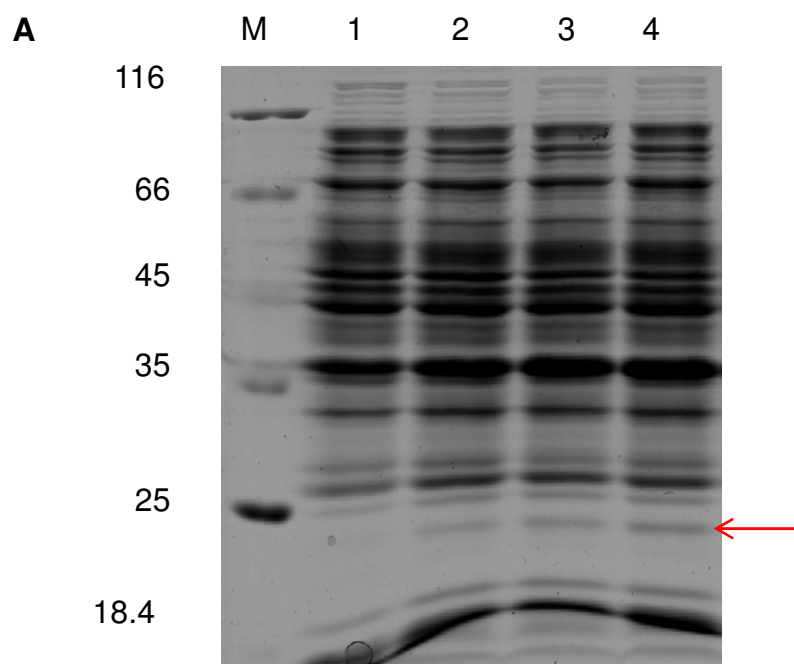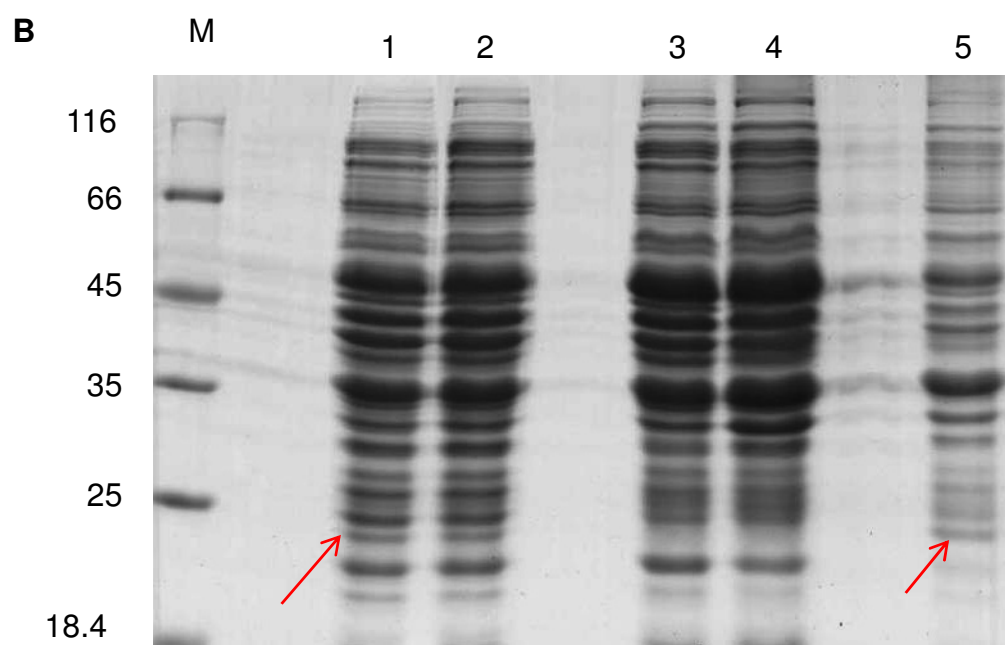

Supplement: Figure S6 — SDS-PAGE analysis of expression of recombinant protein (shown by arrow) in E. coli BL21 (DE3) cells expressing SbSI-2 gene. (A) M marker (kDa), Lane 1: uninduced protein, Lane 2: induced protein 2 h, Lane 3: induced protein 4 h, Lane 4: induced protein 6 h. (B) M marker (kDa), Lane 1 and 2: induced protein 6 h, Lane 3 and 4: uninduced protein, Lane 5: induced protein in liquid assay after 12 h of growth. (PDF) [file pone.0101926.s006.pdf]
